# From Gene to Plate: Molecular Insights into and Health Implications of Rice (*Oryza sativa* L.) Grain Protein

**DOI:** 10.3390/ijms26073163

**Published:** 2025-03-29

**Authors:** Aravind Kumar Jukanti, Divya Karapati, Violina Bharali, Mahesh Gudla, Srinivas Thati, Suneetha Yadla, Manoj Kumar, Raman Meenakshi Sundaram

**Affiliations:** 1 ICAR-Indian Institute of Rice Research, Hyderabad 500030, Telangana, India; 2 Department of Crop Physiology, School of Agricultural Sciences, Malla Reddy University, Hyderabad 500043, Telangana, India; 3 Regional Agricultural Research Station, Acharya NG Ranga Agricultural University, Maruteru 534122, Andhra Pradesh, India; 4 Agricultural Research Station, Agriculture University, Kota 324001, Rajasthan, India

**Keywords:** rice, grain, protein, amino acid, QTL, health

## Abstract

Rice is a staple food crop widely consumed across the world. It is rich in carbohydrates, quality protein, and micronutrients. The grain protein content (GPC) in rice varies considerably. Although it is generally lower than that of other major cereals, the quality of protein is superior. GPC and its components are complex quantitative traits influenced by both genetics and environmental factors. Glutelin is the major protein fraction (70–80%) in rice. Rice protein is rich in lysine, methionine, and cysteine along with other amino acids. Globally, Protein–Energy Malnutrition (PEM) is a major concern, particularly in Asia and Africa. Additionally, non-communicable diseases (NCDs) including diabetes, cancer, cardiovascular diseases, hypertension, and obesity are on the rise due to various reasons including changes in lifestyle and consumption patterns. Rice plays a very important part in the daily human diet, and therefore, substantial research efforts focus on the genetic characterization of GPC and understanding its role in the prevention of NCDs. The contribution of both rice grain and bran protein in improving human health is an established fact. The present study summarizes the different aspects of rice grain protein including its variability, composition, factors affecting it, and its industrial uses and more importantly its role in human health.

## 1. Introduction

Rice (*Oryza sativa* L.) is a major source of food and nutrition for a significant part of the human population by providing nourishment for ~3.5 billion globally [1]. It is cultivated across diverse ecologies in different countries and continents. Globally, rice is cultivated in ~165 m ha, and China and India together account for ~46.0% of the production area [2]. Asia leads in both the production and consumption of rice, and 55% of the production is accounted by China and India [1]. Rice is an important part of the daily diet, and among cereal crops, rice alone provides up to 20% of the recommended daily calorie intake for the global population. Generally, milled rice consists of about 75–78% starch and 5.0–8.0% grain protein while bran contains 10–15% grain protein [3]. Although rice has a relatively lower quantity of protein in comparison to other cereals like wheat and maize, the protein quality of rice is superior with a balanced amino acid profile. Despite the lower protein content, the contribution of rice to overall protein intake is relatively high because large amounts are consumed by billions of people [4].

Globally, nutrient deficiencies and malnutrition are major health concerns. They mostly affect women of reproductive age, lactating mothers, preschool children, and underprivileged communities [1]. Inadequate intake of essential nutrients like protein and others can lead to serious health problems like stunted growth, wasting, underweight, bone issues, and a weakened immune system causing infections especially among the vulnerable population [5]. However, with the burgeoning population and changes in the lifestyle, the demand for dietary protein is increasing globally [6]. The major challenge today is not only meeting the protein demand, but also providing a good quality, cheap, and sustainable supply of protein. Animal protein from poultry, meat, eggs, and dairy have an optimal amino acid composition and high digestibility, making them attractive sources [6]. However, environmental concerns [7], coupled with the association of animal-based diets with an increased risk of cardiovascular disease, diabetes, various cancers, and premature mortality, are some of the serious issues related to the production and consumption of animal protein [8,9,10,11,12].

Heightened health awareness among the consumers has resulted in a significant increase in the consumption of alternative plant-based proteins, mainly for health and environmental reasons [6]. Several efforts are underway to find ‘novel’ and affordable sources of plant protein including the prospect of using waste streams like fruit pomace [13]. However, the best viable sources of dietary protein are plants, especially cereals, pulses, nuts, and vegetables. Globally, the protein intake/supply from plants is highest in Asia (58.83 g/capita/day) followed by Africa (50.61 g/capita/day) [14]. Researchers have extensively studied plant-based diets for their impact on human health, specifically regarding cardiovascular diseases, cancers, diabetes, obesity, and hypertension [6]. Further, interest on the effects of protein-rich foods on various aspects of human health has increased significantly in the recent past [15,16]. Based on the extent of area, and production coupled with its wide geographic habitat and consumption patterns, rice is an excellent source for plant protein.

Therefore, a better understanding of the trait, i.e., protein content, and the development of superior rice varieties would aid in addressing sustainable development goals (SDGs) that include food security and alleviating levels of hunger and malnutrition [17]. Thus, identification of novel sources, genomic regions influencing protein content, and the development of protein-enriched, high-yielding rice varieties are very important. These improved varieties could be a valuable source for nutritionally enriched foods that could aid in both food and nutritional security. In this context, this review presents a holistic view of rice grain protein, discussing its various aspects, genetic regions influencing the GPC, and its implications on human health.

## 2. Protein–Energy Malnutrition (PEM)

**Figure 1:**
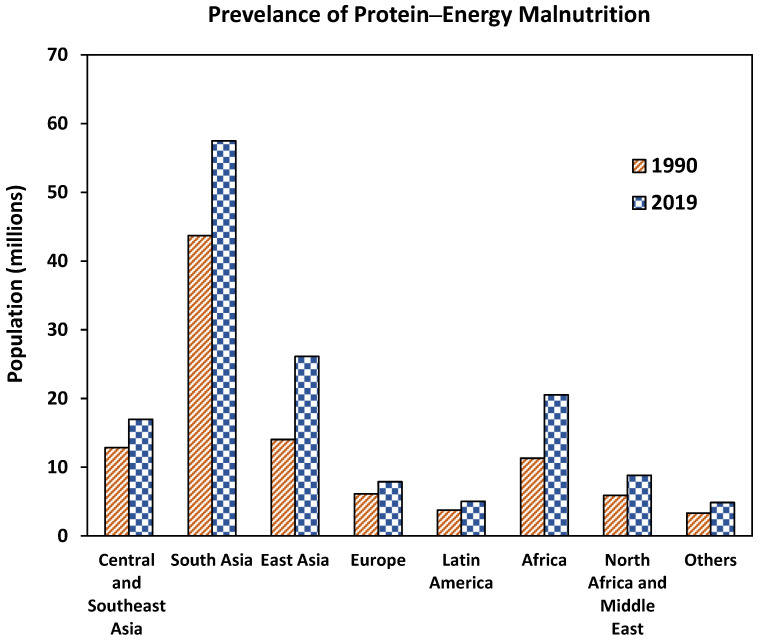
The global prevalence of Protein–Energy Malnutrition (PEM). Others include Oceania, Caribbean, Australasia, high-income Asia Pacific, and high-income North America. Source: [19].

PEM is a universal nutritional problem but mostly reported in Asia and Africa. The prevalence of the PEM varies based on region, gender, and rurality [18]. Globally, PEM affects about 150 million people, and Asia accounts for about 65% of PEM cases followed by Africa [19]. Figure 1 presents the prevalence of PEM across different regions of the world. Though overall, PEM continues to increase, the number of children with PEM is declining globally but varies across regions, viz., PEM among children continues to increase in Africa while a decline in Asia is observed [20]. Despite the advances in medicine and health, PEM still causes unescapable health burdens for all age groups especially in children and elderly by impairing the immune response, which can result in death [18,21]. Addressing PEM should be a top priority as it is a huge public burden.

## 3. Protein Sources, Constitution, and Chemistry

### 3.1. Available Protein Sources

Protein is available in different edible forms in human diet like plant, meat, dairy, seafood, synthetic forms, etc. Plant sources supply the highest range of protein followed by meat and dairy products. Generally, protein provided by animal sources is preferred, but due to increased cost, limited supply, and susceptibility to climate change animal protein is increasingly substituted by plant-based protein. The increasing consumption of plant protein is driven not only by the disadvantages of animal-based diets but also by the numerous benefits of plant proteins themselves. Plant proteins are rich in essential amino acids that are easily absorbed by the body, as well as fiber, polyunsaturated fatty acids, oligosaccharides, and more. Different sources of plant-based protein are cereals, legumes, oilseeds, nuts, vegetables, fruits, etc. Among the different available sources of protein, the cultivation of cereals are under different climatic and geographical regions across the globe (Table 1). Rice is the most important cereal in Asia and Africa, while it is wheat in Europe and Asia coupled with maize in the Americas. Further, the per capita availability of major cereal food crops also follows a trend similar to their production (Table 2). Different sources of protein are predominant in different regions across the globe depending on its preference and availability (Table 3). Plants including the major food crops are the major sources of protein in Asia and Africa. However, in Europe, the Americas, and other regions, meat and dairy also contribute to significant amounts of protein. The per capita supply of protein for consumption on a daily basis from plants is highest in Asian countries (58.83 g/capita/day) and lowest in the Oceania region (38.58 g/capita/day) followed by others (Table 3). Three major cereals (rice, wheat, and maize) are important sources for plant protein especially in Asia and Africa (Table 4).

### 3.2. Protein Content and Quality in Rice

**Figure 2:**
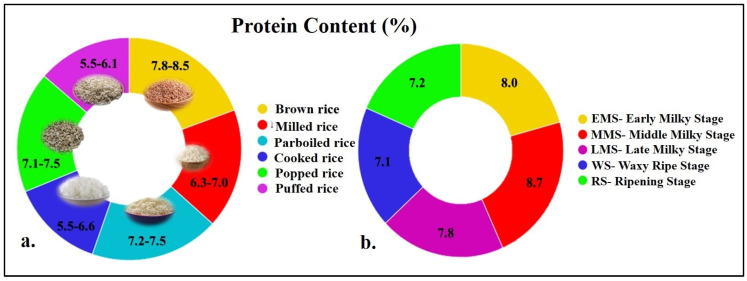
Grain protein content in different types of rice (**a**); grain protein content in different stages of rice grain development (**b**). Protein content is expressed as dry grain weight. Source: [15,24,25,26,27,28,29].

Despite being rich in carbohydrates (75–80%), the grain protein content of milled rice in cultivated rice varieties usually varies between 6 and 8% while in bran it is ~10–15.0% [22,23]. Generally, the protein content in different forms of rice is also around 6–8% (Figure 2a,b) [15,24,25,26,27,28,29]. In addition, the grain protein content in different type of rice varies considerably, viz., indigenous rice (~10–15.0%), land races (5–12.0%), and aromatic germplasm (~11.0%; [30,31,32]. However, the grain protein content in other major cereals like wheat (9–12.0%) and maize (8–12.0%) is relatively higher than in rice [33,34,35,36]. Though the grain protein content in wheat and maize is relatively higher, the qualities of the protein and digestibility are high and the allergenicity is low in rice. Further, the human digestibility of rice is estimated to be >85.0%, while the net protein utilization and bioavailability values for rice [73.8%, 61%] are higher than wheat [53%, 55%] and maize [58%, 61%] [37]. Besides its protein quality, gluten (wheat protein) ingestion causes serious disorders like celiac disease (CD), non-celiac gluten sensitivity (NCGS), and wheat allergy [38]. These disorders can cause an impairment of quality of life and significant morbidity. Maize protein also causes allergies in humans such as skin sensitivities and anaphylaxis [39]. Interestingly, the milky stage of rice grain could be a potential alternative to cattle milk especially for infants owing to its hyperallergenicity and similar constitution to milk casein [29].

### 3.3. Composition and Distribution of Grain Protein in Rice

Based on function there are two major categories of rice proteins: storage and structural proteins [40]. Storage proteins constitute the dominant category in rice seeds. The total content of structural proteins in rice seeds is relatively low but are required for maintaining seed cell metabolism (enzymes, hormones, and enzyme inhibitors) [41]. Therefore, ‘storage proteins’ are considered as ‘rice proteins’. Further, based on the solubility, four sub-categories of storage proteins are present: albumins, globulins, prolamins, and glutelins. Depending on the variety and method of extraction, the content of rice protein fractions varies [22]. The content of albumin, globulin, glutelin, and prolamin also varies significantly in brown rice (5–10%, 7–17%, 75–81%, and 3–6%), milled rice (4–6%, 6–13%, 79–83%, and 2–7%), and rice bran (24–43%, 13–36%, 22–45%, and 1–5%) [22].

Rice-seed storage proteins are mostly present in the aleurone layer and embryo [42]. The most abundant protein form in endosperm (milled rice) is the glutelin while prolamin is equally concentrated in rice bran, fine bran, and milled rice [43]. Albumin and globulin are rich in the aleurone and glume layers. The milling process removes the protein-rich outer layers resulting in milled rice containing a lower protein content compared to brown rice. Endosperm storage proteins mostly occupy the gap between the starch granules as independent protein bodies (PBs). Varieties with a higher grain protein content contain a higher number of PBs compacted and packed around the starch granules. Storage proteins form two types of PBs: (i) PB-I, the ‘spherical type I protein body with concentric sheet structure’ and (ii) PB-II, the ‘ellipsoid type II protein body without sheet structure’ [43]. PB-I and PB-II account for about 20–30% and 65% of the total storage proteins, respectively [44,45]. In PB-I, it is mainly prolamin while in PB-II glutelins are the majority and a few globulins.

#### 3.3.1. Albumins

Albumins are the water-soluble protein fractions of rice accounting for ~5.0% of the seed protein. These are heat liable (denaturation occurs between 73 °C and 75.7 °C), readily digestible, and easily absorbed due to a low number of sulfide bonds. The isoelectric point (pI) of albumins ranges from 6.0 to 7.5, and the molecular weight is between 10 and 200 kDa [22,46]. Albumins in rice bran protein have a lesser molecular weight (<100 kDa) [47]. A family of seven albumin genes are involved in its grain development and quality [48]. Though albumin proteins, RA16 and RA17, are allergenic proteins, they also help in reducing blood sugar and plasma insulin [49,50].

#### 3.3.2. Globulins

Globulins are soluble in dilute salt solutions and constitute ~10.0% of the seed protein in rice [51]. Globulins are rich in sulfur-containing amino acids, cysteine, and methionine. The pI of milled rice globulins ranges 5.9–7.3, and the molecular weight ranges 16 to 130 kDa [22,46]. The reduction in globular fractions resulted in the formation of two polypeptides: 16 kDa (γ-globulins) and 21–26 kDa (α-globulins) [52]. The range of the molecular weight of globulins in bran and brown rice is 10–150 kDa and 23–105 kDa, respectively [22]. Rice contains three globulin genes. The major globulin gene, *Glb-1*, encodes the endosperm-specific, 26-kDa α-globulin and is expressed in the inner endosperm [47,53]. The other globulin genes are *RICE ENDOSPERM GLOBULINs*–*REG-1* and *REG-2* encoding embryo-specific 49-kDa and 46-kDa globulins, respectively [54].

#### 3.3.3. Prolamins

Prolamins are soluble in aqueous alcohol (60–70% ethanol or 50–55% propanol) [46]. The pI of milled rice prolamin ranges from 6.0 to 6.5 [55]. The prolamins consist of three major subunits encoded by at least 34 genes with molecular weights of 10 (pro10), 13 (pro13), and 16 kDa (pro16) [56]; the 13 kDa is the most predominant subunit. The 13 kDa prolamins are classified in two subfamilies: cysteine-rich 13 kDa and cysteine-poor 13 kDa [47]. Among the three subunits, 10 and 16 kDa units are rich in sulfur-containing amino acids, viz., cysteine and methionine [57]. Thirty-four prolamin genes encode the four subfamilies of prolamins [58].

#### 3.3.4. Glutelins

Glutelins are the most abundant storage proteins in rice (70–80%). They are readily soluble in alkaline (pH > 10) or acidic (pH < 3) conditions and are highly heterogeneous [24]. The precursor polypeptide of glutelins has a molecular range of 51–57 kDa [22,59]. The enzymatic hydrolysis of the precursor polypeptide yields two subunits: 19–23 kDa (α/acidic) and 30–40 kDa (β/basic) [22]. The pI of the acidic α subunit is 5.7–5.9, while for the basic β subunit is 8.0–8.7 glutelins in milled rice. A disulfide bond links the acidic and basic subunits resulting in high-order macromolecular complexes explaining the insolubility of glutelins in water [60]. Glutelin is located in the PB II as a crystalloid lattice structure [47]. Glutelin is present in high concentration (66–78%) in the endosperm [61] and contains significant levels of lysine. The rice glutelin family contains at least fifteen genes categorized into four different classes: GluA, GluB, GluC, and GluD [62]. Glutelin type-A (GluA) is further classified into GluA-1 to GluA-4, while glutelin type-B (GluB) includes GluB-1 to GluB-8 [63]. Further, two genes have been identified for GluC and one gene for GluD [43].

### 3.4. Protein Structure

The rice proteins are spherical compared to the irregular polygonal-shaped starches (some starches are incomplete spheres) [29]. Rice proteins also exhibit various basic elements of protein secondary structure, including an α-helix, β-sheet, β-turn, and random coil [52]. Among these, the α-helix, β-sheet, and β-turn have ordered structures, whereas the random coil has a disordered structure [64]. During the progression of seed development, both the molecular weight and secondary structure of fresh rice protein changes [29]. The molecular weight profile progressively changes during the grain development. During the early milk stage (EMS), middle milk stage (MMS), and late milk stage (LMS), peptides between 10 kDa and 25 kDa only were detected. But, at the waxy ripe stage (WS) and ripening stage (RS), proteins with larger molecular weights increased (40 kDa to 130 kDa). Several rice proteins in the molecular weight range from 25 to 90 kDa (α-globulin, glyoxalase 1, protein disulphide isomerase, granulae-bound starch synthase, and α-glucosidase) may act as allergens. But, rice proteins at the milky stage were predominantly below 25 kDa and could serve as promising alternative protein sources. During seed development, the molecular weight of rice proteins becomes larger from the milky stage to ripening stage.

The content of protein secondary structures exhibits varying trends of increase and decrease at different stages of grain development [29]. Both α-helix and β-sheet structures show a gradual increase in their content from the early milk stage (EMS) to the ripening stage. In contrast, the β-turn content decreases from approximately 43.0% at EMS to about 31.3% at the ripening stage. The random coil content rises rapidly from its lowest level at EMS (~9.5%) to its peak value at the middle milk stage (MMS; ~19.4%), before gradually decreasing. Overall, the content of ordered structures increases, while disordered structures decrease from EMS to the ripening stage, leading to more compact and stable protein molecules. The ability to adapt to different conditions including environmental changes varies among the secondary structure of proteins. The environmental changes and processing conditions have a greater impact on β-sheets compared to the other structures [65].

### 3.5. Amino Acid Composition

Amino acids are one of the important nutrients required for growth and development of the human body. They help to regulate physiological activity in the human body by acting as neurotransmitters and humoral factors; and upon oxidation, they provide energy [66,67]. The content of essential amino acids and their bioavailability influences the nutritional quality of protein. Further, the chemical and physical properties of food are to a certain extent defined by the composition of the amino acids [68]. Globally, cereals are a major source of nutrients including protein. Among the cereals, rice is a staple diet for a majority of the population especially in Asia and Africa. Apart from being a source of amino acids, the composition of amino acids in rice protein is balanced and complete compared to wheat and maize [52].

Among the cereals, the rice proteins are ‘easily digestible, have higher biological value with higher protein efficiency quotients’ [52]. Generally, rice contains 18 amino acids including the eight essential amino acids. Table 5 presents the amino acid composition in rice. The content of different amino acids varies between milled and brown rice: (1) histidine: 1.18–3.49% and 1.6–2.6%; (2) isoleucine: 2.69–8.51% and 3.2–4.6%; (3) leucine: 1.68–9.51% and 6.2–8.9%; (4) lysine: 1.28–6.24% and 2.1–4.3%; (5) cysteine: 0.13–3.42% and 1.6–2.4%; (6) methionine: 0.65–3.49% and 2.2–2.8%; (7) phenylalanine: 2.31–6.30% and 4.1–5.3%; (8) tyrosine: 0.81–6.0% and 3.8–6.2%; (9) threonine: 1.98–5.06% and 2.8–4.0%; (10) tryptophan: 0.8–1.8% and 1.1–1.5%; and (11) valine: 2.53–6.80% and 4.3–6.6% [22,24,51,69,70,71,72]. Among the protein fractions, lysine content is highest in albumin followed by glutelin, globulin, and prolamins. Further, rice bran is rich in albumin compared to the milled or brown rice thereby containing a higher lysine content. Interestingly, albumin also has the highest content of sulphur-containing amino acids while prolamin has the lowest [52]. Hence, the highest biological value was estimated to be in albumin while in prolamin the lowest [22].

### 3.6. Surface Hydrophobicity

The surface hydrophobicity is the key indicator for the properties and distribution of hydrophobic residues or hydrophobic amino acids on the surface of proteins [52]. Surface hydrophobicity is considered as a non-covalent interaction between the ligands and is correlated with stability, solubility, foaming, emulsifying, digestibility, and gelatinization properties and protein folding [22,73]. This binding ability between the polysaccharides and protein allows a significant modification in the texture and mouthfeel of food [74]. As discussed above, the predominant constituent of rice protein, glutelin, largely determines the properties of rice protein. The content of hydrophobic amino acid residues in rice glutelin in *O. sativa*, *O. sativa japonica*, and *O. sativa indica*, is 37.2%, 39.6%, and 38.2%, respectively [75]. These can cause hydrophobic interactions among the amino acids, forming network structures between the subunits, thereby restricting the entry of water molecules and reducing the water solubility [75].

The increase in hydrophobicity from the early milking to mid-milking stage in rice is caused by the minimal aggregation and possible exposure of hydrophobic regions buried inside the globular protein [29]. Further, molecular weight variation in the mature grain allows the folding and improvement of the spatial structure of protein with gradual entrenching of the hydrophobic residues into the inner structure, thereby reducing hydrophobicity [29]. Likewise, other factors like oxidation and heat denaturation can alter the hydrophobicity. The lower level of oxidation causes structural unfolding by transforming the β-turn into β-sheets, which causes an increase in hydrophobicity [48]. The heat treatment increased the surface hydrophobicity with the heat-induced intramolecular disulfide linkages forming the interaction between prolamins and glutelins. These interactions cause the conversion of ordered to disordered structures enhancing the increase in the appearance of hydrophobic residues, and thus an increase in hydrophobicity [52,73].

### 3.7. Impact of Changes in Protein Structure

#### 3.7.1. Solubility

Protein solubility is very important in the food industry because it impacts the functional properties of proteins, like emulsifying, foaming, thickening, and gelling [76]. Therefore, insoluble proteins are difficult to use in food applications without changing their structure. Higher solubility of a rice protein indicates a greater exposure of hydrophilic amino acids, which interact well with water molecules. This reduces the hydrophobicity of rice protein [77]. Both temperature and pH impact protein solubility, with temperature being directly proportional to solubility. Higher temperatures help in extracting small oligomers, while lower temperatures release large aggregates of rice protein [51]. Peptides with small molecular weights are more soluble compared to macromolecular peptides [29]. Fresh rice proteins at the milk stage of grain development have a higher percentage of smaller peptides, while, at the ripening stage, they contain a higher content of macromolecular peptides. A lower solubility of rice protein is observed at pH 5, but, at pH 2, the solubility is more than 55%. Glutelin is primarily responsible for a lower aqueous solubility. The lower solubility of glutelin is due to substantial aggregation, hydrophobic interactions, and cross-linking through disulfide bonds [73]. Glutelin from milled rice has a solubility of 8% at pH 5, which can increase to 16% at pH 2 and 30% at pH 8. Further, reducing the molecular weight and increasing the ionizable groups helps to increase the rice protein’s solubility [29].

#### 3.7.2. Foaming Capacity (FC) and Foaming Stability (FS)

The FC and FS of protein are associated with their ability to reduce interfacial tension and are closely tied to the protein’s structure [78]. Therefore, they can be used in constructing consistent films, as surfactants, and to stabilize foams [76]. High temperatures denature the protein structure, causing degradation and disaggregation, which enhance both FC and FS. During denaturation, particles accumulate due to protein unfolding, resulting in improved foaming properties [79]. The FC and FS of a pulse electric field (PEF)-treated rice protein was increased by 2.48 to 2.85-fold and 1.79 to 1.83-fold, respectively. This could be because of changes in the secondary structure from random coils to α-helices and β-sheets [80]. The hydrophobic nature of rice protein and FC are positively correlated, and the hydrophobic regions interact with water molecules [52]. Li et al. [29] reported that the FC and FS values of fresh rice protein at five different stages of grain development vary under different pH concentrations. Both FC and FS decreased and reached a minimum at pH 4 (isolelectric point) and then increased with an increase in the pH. The solubility of rice proteins increased with increase in pH, accelerating the formation and stability of foam [29].

#### 3.7.3. Emulsifying Capacity and Emulsion Stability

The emulsifying activity index (EAI), emulsifying capacity (EC), and emulsion stability (ES) are referred to as ‘emulsifying property’. The emulsifying properties of rice proteins depend on the hydrophobicity, hydrophilicity, solubility, and surface charge of the protein [52]. Due to low water solubility and high disulfide bonds, rice protein has weak emulsifying activity [81]. pH plays an important role in emulsifying properties and is directly proportional to them. Both alkaline and acidic pH improve protein solubility, which causes the breaking of disulfide bonds [82]. During seed development, the emulsifying properties of rice proteins increase with the progress in seed maturation [29]. The EAI increased from 64.73 m^2^/g at the early milk stage to 77.56 m^2^/g at the ripening stage. Further, PEF improved the EC and ES of rice proteins by 3.3–5.3% and 9.6–12.0%, respectively, compared to the alkaline treatment [80]. This could be due to the improvement in protein structure, i.e., ordered structure increases thereby resulting in the increase in emulsifying capacity.

#### 3.7.4. Protein Digestibility

Consumption of rice-based products with high protein digestibility could improve health and nutritional status. The secondary structure of protein, hydrophobicity of protein, and protein cross-linkings (e.g., disulfide cross-linking) are important factors that affect protein digestibility [83]. Rice protein fraction prolamin accumulates in the type-I protein body (PB-I; 20–30% of rice storage proteins) while glutelin and globulin accumulate in the type-II protein body (65% of rice storage proteins) [44]. The strong hydrophobic nature facilitates disulfide bond formation between the polypeptides of prolamin; PB-I particles exhibit a consistent, round, spherical morphology and are highly insoluble in water [43]. Due to this insoluble and indigestible character of rice prolamin, the properties of PB-I do not change after cooking, exhibit a strong resistance to proteolytic enzymes, and is not absorbed by the human body [45]. In contrast, glutelins and globulins, which constitute PB-II, are soluble proteins with weak physical properties, making them easily digested and absorbed. But, alkali treatment for the extraction of rice protein improved protein digestibility and bioavailability and reduced the fecal excretion of prolamin [45]. This could be due to the degradation or loosening of PB-I by alkali treatment; prolamin is digested and absorbed rather than being excreted into feces. 

## 4. Factors Influencing Grain Protein Content in Rice

### 4.1. Impact of Temperature

Ecological (temperature, carbon dioxide concentration, and light) and cultivation practices affect the grain protein content in rice. GPC is highly sensitive to an increase in temperature during the grain-filling stage [40]. High temperature during the grain-filling stage in rice leads to an increase in protein content, but an overall decrease in grain quality [84]. An average rise of 5 °C in the air temperature at grain filling in rice increased the GPC by 21% [85]. Further, a rise of 1.6–3.1 °C of the air temperature at grain filling changed the protein composition in rice: a reduction in prolamins by 12% and an increase in glutelin content by 31% [73]. High temperature at the mature stage leads to an abnormal rice quality affecting its shape and color [40]. In addition to the temperature, low-light treatment increased the content of glutelin and two important amino acids (lysine and threonine) significantly [86].

### 4.2. Impact of Carbon Dioxide (CO_2_)

Studies have shown that the CO_2_ concentration affects the rice grain quality. Increase in atmospheric CO_2_ leads to a decrease in the rice’s GPC, which could be due to the inhibition of nitrate assimilation [87]. The GPC of rice decreased by 7.0% at CO_2_ exposure of 500 ppm [88]. Elevated synthesis of carbohydrates or reduction in leaf protein content could also be the reasons for the decrease in the GPC [88,89]. Although GPC decreases under the elevated CO_2_ levels, the overall grain protein yield per hectare may not reduce, as it is compensated by an overall increase in grain yield per hectare [90]. However, elevated CO_2_ concentration changes the viscosity parameters thereby enhancing the cooking and eating quality of rice [87].

Interestingly, the elevated CO_2_ levels will also affect the protein composition and amino acid content. Jing et al. [91] exposed the rice cultivar to 200 ppm above the ambient concentration of CO_2_ from tillering until maturity resulting in significant changes in the protein composition. The albumin, prolamin, glutelin, and globulin showed a reduction by 34%, 21%, 17%, and 16%, respectively, under elevated CO_2_ conditions. An increase in CO_2_ levels by 500 ppm has led to a reduction in the content of several amino acids, including threonine (a decrease of 1.6%), valine (decrease of 4.5%), methionine (decrease of 5.0%), isoleucine (decrease of 1.9%), leucine (decrease of 1.7%), phenylalanine (decrease of 1.5%), and lysine (decrease of 2.6%) [88]. The combined stress of a higher temperature (by 5 °C) and elevated CO_2_ levels (by 700 ppm) at the reproductive phase resulted in a reduction in GPC by 4.0 to 6.0% compared to elevated CO_2_ alone [92].

### 4.3. Impact of Management Practices

The two most important factors in cultivation management are water and fertilizer. Different water management strategies like mulching, grouting, and conventional irrigation significantly influence the grain quality including the GPC based on the position of the grain and variety. The effect of water management is larger on the GPC compared to other quality traits [93]. Water treatment accounted for 43.2% of the phenotypic variance of GPC while the interaction of the water treatment by genotype and grain position accounted for 27.8% and 13.6%, respectively. The GPC was significantly higher in the top grains of the spike compared to the bottom ones. An average increase in GPC (0.5%) and grain yield (0.6 metric tons/ha) was recorded under continuous flooding compared to intermittent irrigation [94]. Nitrogen availability is another critical factor that influences plant growth and development. Nitrogen is the main constituent of grain protein, and therefore the application of nitrogen fertilizers can have a substantial effect on the GPC and quality [95]. The timing of the application of nitrogen is important as its application at heading, flowering, and grain filling could increase the GPC [63]. Interestingly, some studies have shown that even application of potassium fertilizer increased GPC [40].

### 4.4. Impact of Stress Conditions

Drought and salinity conditions also affect the GPC in rice. Both the protein content and grain yield of rice reduced under water stress conditions [94]. The reduction could be due to a decrease in N uptake since water stress or soil aeration, or both, affected the N availability to the plant. The GPC of rice grown in puddled soils was higher in comparison to the rice grown in non-puddled soils [96]. Salinity significantly affects the crop quality. Both salt-tolerant and salt-sensitive varieties grown in saline soils had a higher GPC compared to those grown in normal soils. However, these varieties had less translucent grains coupled with a lower starch and amylose content [97].

### 4.5. Impact of Post-Harvest Processing

The hulling/de-husking of rice removes the husk-yielding brown rice. However, milled rice obtained after the removal of the bran layers (pericarp, seed coat, and aleurone layer) is consumed [98]. Unfortunately, milling can cause a significant loss in nutrient content including GPC, as different nutrients are concentrated in the embryo and bran layer [99]. The degree of milling (DOM) affected the rice composition including the GPC [98]. At first milling stage (2 s), when the degree of milling was ~3.0%, the protein loss was about 40% of the total loss rate indicating the highest concentration of GPC in the pericarp and seed cortex layers of the rice bran. The GPC and its composition also showed a reduction at different milling stages from 2 to 14 s.

## 5. Genomic Regions Affecting Grain Protein Content, Protein Fractions, Amino Acids, and Protein Index

GPC and quality traits are polygenic/quantitative traits as multiple genes or multiple loci affect them. Additionally, the environment also significantly affects GPC. Hence, it is difficult to manipulate GPC through traditional breeding approaches because most of the variation inherited is quantitative in nature [100]. Therefore, identification of resources (for GPC and quality traits) that can be used in the identification of genomic regions/QTLs that are associated with traits of interest is of the utmost importance. Hence, improvement in GPC and amino acid content is possible by using multiple approaches including QTL identification and marker-assisted breeding methods. Usually, quantitative traits like GPC require the pyramiding of multiple QTLs from different sources for significant improvement [101]. Researchers have identified several QTLs and a few candidate genes for total protein content, amino acids, protein fractions, and protein index (Supplementary Table S1).

### 5.1. QTLs Identified for GPC and Protein Components in Rice

The distribution of QTLs identified for GPC and other component traits spans all twelve chromosomes in rice (Supplementary Table S1). Researchers have identified more than 40 QTLs that affect GPC, its components, and amino acids (Supplementary Table S1). The identified QTLs are from brown rice, milled rice, and aromatic rice. Some GPC QTLs identified co-located with other traits like different amino acids [102]. Interestingly, co-localization and pleiotropism of heading-date genes on GPC and amino acid content is also reported [103]. Another GPC QTL, *qPC7*, is at the same flanking marker as amylose content QTL, *qAC7* [104]. Further, a QTL cluster on chromosome 8 near G1149 marker included nine QTLs for different quality traits including the following: percentage of grain with chalkiness (PGWC), area of chalky endosperm (ACE), amylose content (AC), protein content (PC), breakdown viscosity (BDV), setback viscosity (SBV), cooked rice luster (LT), tenderness (TD), and viscosity, elasticity, and the integrated values of organleptic evaluation (IVOE) [105]. More importantly, this QTL cluster exhibited stability and repeatability across eight environments.

Three independent studies identified three QTLs that co-localized with the *Waxy* locus [106,107,108]. *qPC-6* which is located in the *Wx* region [106] was detected from an intra-subspecific cross [109], inter-subspecific cross [108], and inter-specific cross [107]. Similarly, other QTLs, *qGP6*-1 and *qPC-6*, were detected near the *Wx* locus from the intra-specific crosses [110,111]. Yu et al. [106] identified two other QTLs, *qPC-3* and *qPC-4*, which were near to a QTL for GPC in brown and polished rice [112] and a QTL for GPC in polished rice [108,112], respectively. The QTLs identified by [107], especially *qPC-3* and *qPC-4*, could be useful in improving the nutritional quality, in addition to GPC as these are not associated with any adverse effects on other traits. *qPC12*-affecting crude protein (CP) co-located to the same region as *qGTL-12*, affecting glutelin content [113]. Further, in agreement the CP and glutelin content had a positive correlation. In a recent study, two protein QTLs, *qseqPC2.1* and *qPC2.1*, identified using BSA-seq analysis and QTL analysis, respectively, were associated with a high amylose and low GI content QTL *qGI2.1/qAC2.1* leading to the development of nutritionally rich rice [114]. The QTL *qseqPC2.1* overlapping *qseqAC2.1* consists of glutelin-related genes and genes involved in sugar transport (LOC_Os02g13560—*tonoplast monosaccharide transporter 2*) and meiotic recombination during fertilization (LOC_ Os02g13810—*Human enhancer of invasion 10*). Further, *qseqPC2.2* overlapping with *qseqAC2.2* and *qAC2.1* includes genes like *OsSBEIIb*, *OsNIN3,* and *OsCIN1*, as well as candidate genes encoding glutelin, *Glutelin A*. Interestingly, a single nucleotide change to the homozygous A allele in *SBEIIb* accounted for 57.2%, 60.0%, and 8.0% of the PVE in GI, AC, and 8%, respectively. Several studies also identified QTLs for individual amino acids (Supplementary Table S1). He et al. [115] identified a genic region for lysine biosynthesis, *LOC_Os07g20544* encoding the Asparto kinase (AK) protein. The AK gene carried three haplotypes: Hap1 (GGCCGGAATTTTGG, *n* = 314), Hap2 (CCCCAACCCCCCAA, *n* = 41), and Hap3 (GGAAGGAATTTTGG, *n* = 27). Interestingly, a significantly lower lysine content was observed in accessions carrying Hap2 compared to those having Hap1 and Hap3. Further, analysis of the transcription factor (TF) binding site identified a WRKY DNA domain-binding domain containing a protein. Haplotype analysis of this WRKY TF revealed a significantly higher lysine content in accessions with Hap1 (GGTT, *n* = 254) compared to those with Hap2 (AACC, n-131). A total of 73 QTLs for 17 amino acids were detected, among them 5 QTLs for lysine (*qLys1.1*, *qLys1.2*, *qLys6*, *qLys9*, and *qLys12,*), 3 for cysteine (*qCys7*, *qCys9*, and *qCys12*), and 3 for methionine (*qMet6*, *qMet9*, and *qMet11*) [103]. The largest cluster was located in the RM190-RM6917 region on the short arm of chromosome 6, which has two florigen genes (*RFT1* and *Hd3a*). Jang et al. [102] identified 17 QTLs for amino acid content (AAC). Among the 17 QTLs for AAC, Jang et al. [102] reported *qAAC6.1* and *qAAC7.1*, which are associated with more than 10 AACs, for the first time. In addition, the allelic combination of *qAAC6.1* (M23), *qAAC7.1* (T887), and *M23-qAAC7.1T887,* significantly increased the amino acid content compared to others. Further, *M23-qAAC7.1T887* exhibited higher GPC and amino acids (methionine, histidine, lysine, and glycine) than both the parents did. However, Jang et al. [102] did not find any QTL for proline. An interesting study by Yoo et al. [70] detected six main-effect QTLs (M-QTLs) for six amino acids (alanine, valine, leucine, isoleucine, phenylalanine, and lysine) along with 26 epistatic QTLs (E-QTLs). The E-QTLs explained far greater phenotypic variation for traits than M-QTLs, demonstrating that epistasis plays a significant role in controlling the expression of AAC. Breeding programs need to consider the epistatic effect of the E-QTLs when utilized.

A study of 188 F_9_ recombinant inbred lines (RILs) derived from a cross between Zhenshan 97B and Delong 208 yielded 48 and 64 QTLs during 2004 and 2005, respectively [116]. Majority of these QTLs co-localized and formed 29 QTL clusters. Among the 29 clusters, three major clusters were detected on chromosomes 1 (*qAa1*), 7 (*qAa7*), and 9 (*qAa9*) during both the years for AAC. *qAa1* and *qAa7* influenced almost all the traits with the allele from Zhenshan 97B while *qAa9* increased the lysine content with the allele from Delong 208. The QTLs detected co-occurred with the loci involved in amino acid metabolism pathways in nitrogen assimilation and transport, or protein biosynthesis. Interestingly, another study of 190 RILs identified 18 QTL clusters, of which 12 corresponded to loci involved in amino acid metabolism [117]. Two major QTL clusters between RM472-RM104 (1–19) and RM125-RM542 (7–4,5) were detected in both the years of study. A few of these 18 QTLs co-mapped with more than one locus in amino acid metabolism. RM125-RM542 (7–4,5) co-mapped with storage proteins including globulins, albumins—while RM315-RM104 located on chromosome 1 mapped with genes encoding AATs—and aspartate kinase (involved in aspartate family amino acid biosynthesis). The QTL clusters in RM322-RM521 (2–7) co-localized to a region having Asp-AT4 and three members of a glutelin subfamily (GluB-1, GluB-2, and GluB-4).

From two BC_3_F_2_ populations, ref. [101] detected 18 and 14 QTLs in P-I and P-II, respectively, for all four protein fractions and are observed to be co-localized with previously reported QTLs. Fiaz et al. [111] identified a total of 44 QTLs, protein content (5), glutelins (8), globulins (10), albumins (9), and prolamins (9) and observed that *qGLU6, qPRO6,* and *qALB6* were co-localized with *qPC6*. Among the protein QTLs, *qPC6* was detected under all of the three environment conditions. The *qPC6*, *qGLU6*, *qPRO6*, and *qALB6* alleles were inherited from *Indica* YK17, and they decreased the content of protein, glutelin, prolamin, and albumin under all of the three growing environments. Further, *qPC6* spanned the *Wx* gene region, suggesting that *qPC6* may correspond to the Wx gene. *qGLU6* was consistently detected in the same genomic region as *qPC6*, with a negative additive effect contributed by Indica YK17. Furthermore, a highly significant correlation (r = 0.5526 **) between protein content and glutelin was observed. These results suggest that PC and GLU may share the same genetic mechanism. Zhang et al. [113] identified 16 QTLs for different protein fractions. Some of these QTLs affecting different protein fractions are located in the same chromosomal region. A major QTL, *qPLA-10*, for prolamin content was co-mapped with *qGLT-10. qGLT-10* has the largest effect on glutelin content, corroborating a highly positive correlation between prolamin and glutelin contents. These findings suggest that glutelin and prolamin contents could partially share the common genetic mechanism [53]. However, it is possible to identify separate alleles for glutelin and prolamin as the majority of the QTLs for these fractions are on different chromosomes. Another glutelin QTL, *qGLT-2*, mapped in the same region as *qGLB-2.1*, a QTL for globulin content. Interestingly, *qCP-12*, which accounts for 14.0% of phenotypic variation in CP, mapped to the same region as *qGLT-12*, a QTL for glutelin content.

QTL for protein index (milligrams of protein per milled rice; PI), a property calculated from PC (100 kernel weight X 1000 X PC/100), was identified [118]. Among the eight QTL identified across three environments, *qPI-3.1* and *qPI-7* were identified across all the three environments, while *qPI-3.2* in two environments, and others were detected only in one environment. *qPI-6.1* resided in the interval R1952-G200 close to both *Wx* and *Alk* loci, while *qPI-6.2* located near to *Alk* locus. Further, *qPI-7/qPC-7.2* co-localized in the same interval C847-C596, qPI-3.2/qPC-3 to R250-C746, and qPC-10/qPI-10 to C16-C809, consistent with the highly significant positive correlation between PC and PI.

### 5.2. Candidate Genes Controlling GPC and Protein Components in Rice

Aspartate aminotransferase (AAT) is a key enzyme in the synthesis of amino acids, and it plays an important role in regulating carbon and nitrogen metabolism. Zhou et al. [119] over-expressed separately three AATs (*OsAAT1*, *OsAAT2*, and Os*AAT3*) and an *Escherichia coli* AAT (*EcAAT*) in rice. *OsAAT1*, *OsAAT2*, and Os*AAT3* localize to the chloroplast, cytosol, and mitochondria, respectively. The AATs’ activity in the leaves of plants overexpressing *OsAAT1*, *OsAAT2*, and *EcAAT* was 26.6, 23.6, and 19.6 A/min/mg FW, respectively, which were considerably higher than that in the control (17.7 A min^−1^ mg^−1^ FW). Transgenic plants overexpressing *OsAAT1*, *OsAAT2*, and *EcAAT* had 22.2, 21.1, and 11.1%, respectively, higher protein contents than wild-type plants. In addition, the amino acid content in the seeds of plants overexpressing *OsAAT1*, *OsAAT2*, and *EcAAT* was 119.36, 115.36, and 113.72 mg g^−1^, respectively, 16.1, 12.0, and 5.4%, respectively, higher than that in the control plants. The contents of all 17 amino acids, except for Glu in *OsAAT1*-overexpressing plants and Cys in *OsAAT2*-overexpressing plants, were significantly increased (ranging from 10.3% to 39.1%) compared to the negative controls. This suggests that the overexpression of these genes could be an effective strategy for enhancing the nutritional quality of rice grains. Interestingly, the overexpression of *OsAAT1* (encodes chloroplast AATs) and O*sAAT2* (encodes chloroplast AATs) increases the amino acid content in grains and AATs’ activity in leaves, but no significant changes were observed when *OsAAT3* (encodes mitochondrial AATs) was overexpressed. The very high expression levels of the endogenous *OsAAT1* and *OsAAT2* compared to *OsAAT3* in WT plants suggest that these two genes play an important role in the transfer of the amino group in rice plants.

Reduced dependency of plants on N fertilizer and improving their nitrogen use efficiency (NUE) are urgently required for environmental and agricultural sustainability [120]. This is especially important for rice, which is cultivated with a high input of N fertilizer and serves as a major food staple, providing 21% of global human per capita energy and 15% of protein. Lee et al. [121] reported a rice asparagine synthetase 1 (*OsASN1*) that is required for grain yield and protein content under both N-sufficient and N-limiting conditions. Under field conditions, in the *OsASN1*-overexpressing plants (OX1 and OX2), N content increased to 140% and 131% compared to that in the control, respectively. Researchers observed a 3.4- and 3.1-fold higher level of asparagine in the grain of OX1 and OX2 plants, respectively, compared to the control plants. Grain protein concentration of both OX lines was elevated to 120% compared to that in the control and free amino acid concentrations to 127% (OX1) and 113% (OX2) compared to the control. Similarly, under N-limiting conditions, the N content of OX1 and OX2 increased to 132% and 133%, respectively, over the control, while the grain protein content increased to 117% (OX1) and 114% (OX2) compared to the control.

In contrast to *osasn1* mutants, *OsASN1* OX lines showed an increased nitrogen uptake and influx. The enhanced expression of *OsGGS1;1* and *OsGOGAT1* indicated that nitrogen assimilation through the GS/GOGAT cycle was more active in the *OsASN1* OX lines than in WT plants, resulting in a higher ammonium incorporation into amino acids. As a result, it is hypothesized that the OX lines increased asparagine synthesis in their leaves, leading to an enhanced nitrogen and protein allocation to their seeds. However, no significant changes in plant height, biomass, or yield were observed in conventional paddy fields. This suggests that *OsASN1* overexpression has the potential to improve GPC without affecting grain yield under conventional agricultural practices. Under nitrogen-limiting conditions during the reproductive stage, the *OsASN1* OX lines exhibited an enhanced photochemical efficiency in their leaves compared to WT plants, resulting in better performance, increased biomass, grain filling, and yields in the OX lines compared to WT plants. Overexpression of *OsASN1* leads to improvements in grain yield, N, and protein content, in plants grown under N-limiting conditions. Thus, *OsASN1* is an important target candidate for developing high protein rice, improve nitrogen use efficiency, and increase grain yield.

The plant redistributes most of the N supplied to panicles from the leaves, and about 70% of the leaf’s N can be transported to the panicles during grain filling [122]. Amino acids’ import into seeds via the phloem is an important event in source-to-sink N partitioning, and the amount of amino acids translocated mostly decides the sink development and grain yield [123,124]. Researchers have identified, characterized, and manipulated several amino acid allocation proteins. A genetic association analysis between the ^15^N-aspartate uptake rate of rice core accessions and the SNPs of predicted amino acids’ transporter genes identified ‘Lysine-Histidine-type Transporter 1 (*OsLHT1*)’ to be an important transporter for root uptake and the root–shoot allocation of amino acids [125]. Guo et al. [126] studied the knockout mutant lines of *OsLHT1 (Oslht1)* along with wild type (WT) plants for evaluating the function of *OsLHT1* in N redistribution and grain production. *OsLHT1* is expressed in the vascular bundles of leaves, rachis, and flowering organs. Plants with the *Oslht1* showed a reduced panicle length and seed setting rate and lower grain number per panicle and total grain weight. Total N and amino acid concentrations in the mutants were significantly higher in mutant lines compared to WT at maturation compared to anthesis. Total N, protein content, and individual amino acids were accumulated by 30–35% higher in seeds of mutant lines compared to the WT plants. Interestingly, the amylose content in mutants decreased by 31%, whereas the starch content was similar in both WT and mutant lines. Further, in mutant lines the paste viscosity was lower than the WT lines. Therefore, *OsLHT1* is important for the translocation of amino acids from vegetative to reproductive organs affecting grain yield, quality, and nutrition.

Wang et al. [117] identified 18 chromosomal regions for 19 components of amino acid content (AAC), and most of these QTLs were stable over different years of study. The average variation explained by individual QTLs varied from 4.3% to 28.82%. Wang et al. [117] found a relatively strong QTL cluster controlling GPC on the long arm of chromosome 1, consisting of up to 19 individual QTLs encoding putative amino acid transporters (AAPs). A region within RM472-RM104 on the long arm of chromosome 1 explained the highest genetic variation (32.4%) by an individual QTL (*qPC1*) among the cluster. A gene (s) underlying the *qPC1* QTL (a putative amino acid permease, *OsAAP6*) was identified using recombinant inbred lines derived from a cross between Zhenshan 97 (ZS97, *indica*) and Nanyangzhan (NYZ, *japonica*) [127].

The impact of *OsAAP6* on GPC was studied using three transformation constructs: (1) overexpressing (OX) the coding region of *OsAAP6* from ZS97 (high GPC), (2) a complementation construct (ZpZc) with the promoter and coding regions of *OsAAP6* from ZS97, and (3) a RNAi construct containing a 580-bp PCR fragment from the fourth exon of *OsAAP6*. Three transgene-positive plants (OX[NYZ], OX[ZH11] and ZpZc[NYZ]) along with NIL(ZS97) exhibited significantly higher GPC and amylose content coupled with a substantial reduction in starch content and gel consistency whereas a reverse trend was observed in RNAi plants. The protein content in the transgene-positive plants ranged 118.4–124.7 mg g^−1^ (brown rice) and 102.1–107.1 mg g^−1^ (milled rice), whereas, in the transgene-negative, GPC was 106.8–108.7 mg g^−1^ (brown rice) and 92.6 to 95.5 mg g^−1^ (milled rice). A similar increase was also observed for protein fractions (glutelins, prolamins, globulins, and albumins), amino acids (alanine, leucine, valine, proline, and arginine), acidic amino acids, and the total content of amino acids in transgene-positive plants compared with the corresponding levels in NIL(NYZ) and transgene-negative OX(NYZ) plants.

Chattopadhyay et al. [128] has identified several QTLs for GPC, but only three QTLs (*qGPC1.1*, *qSGPC2.1,* and *qSGPC7.1*) were stable across environments. Among the stable QTLs, the *qGPC1.1* interval region corresponded to a span of 186 *O*. *sativa Japonica* genes starting from *Os01G0111600* to *Os01g0119500*. Among the genes located inside these QTLs, one gene *Os01g0111900* (locus-1:625986–627009) encoded a glutelin family protein. Apart from this, several putative genes (*Sar1c*-seed storage protein; *OsAAP6;* glutelin protein; anthranilate synthase alpha 1 and *OsAsp1*) identified were from single environment QTLs.

## 6. Health Benefits of Rice Grain Protein and Its Derivatives

Nutrition plays a crucial role in human health across geographies and societies [129]. It improves the quality of life and helps in being free from non-communicable diseases (NCD) at least for a certain period, in addition to fighting against communicable diseases. Since the role of nutrition in human health is complex, the impact of dietary interventions is less clear, despite several studies by researchers. However, access to quality, nutritious food along with the host’s immunity are primary contributors to a long and healthy lifespan. Globally, NCDs pose a significant health challenge. NCDs are the leading cause of death worldwide, killing about 41 million people each year. The major killers among the NCDs every year are cardiovascular diseases (17.9 million), different cancers (9.0 million), respiratory diseases (3.9 million), and diabetes (1.6 million) [130].

Rice is a major staple cereal crop across the world especially in Asia and Africa. It is an important source of carbohydrates and protein for humans. Further, rice protein exhibits various physiological effects like anti-hyperglycemia, antihyperuricemia, and anti-obesity [131,132,133]. In addition, rice protein is a novel source of functional peptides that are physiologically active [134]. Moreover, studies have shown that rice proteins, both endosperm protein (REP) and bran protein (RBP), are effective against NCDs and other health conditions, which the following sections describe in detail.

### 6.1. Antioxidant Activity

High cholesterol levels in blood plasma could be due to multiple factors. Interestingly, several studies suggest that oxidative stress is one of the major causes of hypercholesterolemia [135,136]. An imbalance between the production and scavenging of free radicals by antioxidants is ‘oxidative stress’ [137]. Dietary antioxidants can control oxidative stress thereby underscoring the role of diet in the prevention of hypercholesterolemia [138]. A three-week study on seven-week-old male Wistar rats demonstrated that rice proteins (RP) significantly reduced the plasma and hepatic total cholesterol (TC) levels by 11.3% and 27.0%, respectively [139]. Further, there was a significant decrease in hepatic tricglycerides (TG) in rats fed with RPs by 35.2% in comparison to those fed with casein. After three weeks of feeding with RP, the accumulation of malondialdehyde (MDA) and protein carbonyls (PCO) decreased substantially by 23.0% and 21.3%, respectively. There was a significant stimulation in the total antioxidative capacity (T-AOC; 27.5%) and reduced glutathione (GSH; 40.9%), while the oxidized glutathione (GSSG) accumulation was decreased (18.0%) in the liver of rats fed with RP. RP feeding enhanced the activity levels of hepatic enzymes involved in antioxidative activities, such as superoxide dismutase (T-SOD) and catalase (CAT), by 60.0% and 12.4%, respectively. Similarly, the activity levels of enzymes involved in glutathione metabolism like glutathione S-transferase (GST), glutamylcysteine sythetase [γ-GCS], glutathione reductase (GR), and glutathione peroxidase (GSHPx) were significantly stimulated in RP-fed rats by 21.6%, 63.4%, 69.3%, and 11.2%, respectively. RP feeding effectively stimulated the mRNA levels of glutamate cysteine ligase modulatory subunit (GCLM) and glutamate cysteine ligase catalytic subunit (GCLC) in the livers of rats. A study demonstrated that RP improved oxidative stress predominantly through antioxidative defense mechanisms; RP improved the antioxidative status and mitigated the oxidative damage to lipids and proteins [139].

Cai et al. [140] has shown that RP exhibits hypocholesterolemic action through antioxidant activity and by reducing the oxidative damage in adult rats. RP in comparison to casein stimulated in liver the total antioxidant capacity (T-AOC) and levels of T-SOD and catalase (CAT) along with γ-GCS, GR, and GSHPx involved in glutathione metabolism. RP also increased the GSH but decreased the accumulations of GSSG, MDA, and PCO. Overall, a significant decrease in cholesterol through antioxidant activity was observed in the plasma and liver of adult rats fed with RP. Li et al. [141] also demonstrated an increased hepatic accumulation of GSH, but decreased content of GSSG and ROS in RP-fed rats. The activity levels of two biomarkers for liver damage, plasma alanine transaminase (ALT) and aspartate transaminase (AST), were significantly depressed by RPs (ALT: −42.53%; AST: −43.92) compared to casein-fed rats. Also, the mRNA levels of GCLC, GCLM, and GST along with heme oxygenase 1 (HO-1) and NAD(P)H:quinone oxidoreductase 1 (NQO1) were significantly increased. RP feeding increased the accumulation of the nuclear fraction of ‘Nuclear factor erythroid 2-related factor 2 (Nrf2)’ under cholesterol-enriched conditions in rats, compared to casein feeding.

Interestingly, the different extraction methods used for RP might also affect the antioxidant capacity of RP [142]. The in vitro antioxidant capacity of RPs by alkali (RPA) and α-amylase (RPE), i.e., protein digests followed by the successive digestion with pepsin and pancreatin (RPA-S; RPE-S), i.e., hydrolysates, differed significantly. Overall, the protein digest, RPE exhibited a strong antioxidant activity to free radical scavenging (1,1-diphenyl-2-picrylhydrazyl, DPPH), metal chelating (Fe^+2^ and Cu^+2^), and reducing power. Wang et al. [142] have shown that protein digests (have undigested residues) probably exhibit more efficient antioxidant activity compared to hydrolysates. In another study by [143], glutelin (RPG) and prolamin (RPP), two major components of RP with different digestibility levels, have exhibited differences in in vitro antioxidant activities. In comparison to RPP, RPG has shown a stronger antioxidant response to the free radical scavenging of 2,2′-azinobis [3-ethylbenzothiazoline-6-sulfonic acid] diammonium salt (ABTS), superoxide radical scavenging, hydrogen peroxide scavenging, nitric oxide (NO) radical scavenging, metal chelation (Fe^+2^ and Cu^+2^), and reducing power. The increased digestibility and availability of amino acids of RPG could probably be responsible for the excellent antioxidant capacity of glutelin compared to prolamin (RPP).

Defatted rice bran (DRB) is a good source for nutritional proteins. DRB hydrolysates (using proteases) and their fractions exhibit antioxidant properties [144]. The protein hydrolysates obtained from alcalase and pepsin–pancreatin digestion exhibited significant antioxidant activity (radical scavenging activity of ABTS, DPPH, and NO). Protease specificity, peptide length, and the amino acid sequence could relate to the antioxidant activity of the hydrolysates. Further, the fractions/peptides exhibiting antioxidant activity were composed of histidine (H), tryptophan (W), and tyrosine (Y). Protein yield from rice bran protein hydrolysates (RBPH) improved significantly upon enzyme hydrolysis with different proteases especially alcalase [145]. Additionally, the purified RBPH fractions from alcalase digestion exhibited antioxidant activity 1.04 to 5.64 times higher than that of the crude hydrolysates. These results indicate that RBPH, derived from alcalase digestion, could potentially be utilized as a nutritional supplement or ingredient in functional foods and beverages.

Another study by Wattanasiritham et al. [146] demonstrated that trypsin-hydrolyzed, denatured albumin had the highest antioxidant activity compared to other native, denatured, non-hydrolyzed or hydrolyzed protein fractions (prolamin, glutelin, and globulin). The ‘Oxygen Radical Absorbance Capacity’ (ORAC) of RBPH–albumin (RBPH-A) was 4.07 μmol of the trolox equivalent (TE)/mg protein. Most of the peptides obtained from RBPH-A had amino acids with intrinsic antioxidant activity especially hydrophobic amino acids at C- and N-terminus. Interestingly, the addition of methionine (an essential sulphur-containing amino acid) to RPs (RMs) augments their antioxidant activity [147]. The hydrolyzed RMs exhibited a significant increase in free radical scavenging (SO; NO) and reducing power compared to RPs. This indicates the importance of the availability of methionine as a critical factor in antioxidant ability of RPs.

### 6.2. Hypocholesterolemic Response

Yang et al. [148] demonstrated a decrease in the plasma total cholesterol (TC) concentration and an increase in high-density lipoprotein–cholesterol (HDL-C) to TC by ~12.4% and ~18.2%, respectively, in rats fed with RPs compared to those with casein. Further, the reduction in liver lipids could be due to a decrease in hepatic free- and esterified cholesterol levels by ~17.8% and ~35.6%, respectively. Interestingly, RPs effectively increased cholesterol 7α-hydroxylase (*CYP7A1*) by ~46.1%, a limiting enzyme in the conversion of cholesterol to bile acids; RPs reduced the enzymatic activity of hepatic acyl-CoA: cholesterol acyltransferase-2 (ACAT-2), an important regulator for cholesterol output and cholesterol absorption. In addition to the regulation of CYP7A1 and ACAT-2, adult rats fed RPs revealed considerable stimulation of hepatic gene expression levels of the liver X receptor α (LXRα) and peroxisome proliferator-activated receptor α (PPARα), other important enzymes involved in cholesterol metabolism [149].

In another study involving adult male Wistar rats, RPs reduced the TC and triglyceride (TG) levels in the plasma by ~15.7% and ~23.6%, respectively, in comparison to casein, thereby resulting in a significant increase in HDL-C to TC and HDL-C to TG by ~22.7% and ~31.6%, respectively [150]. In liver, the RPs significantly reduced the TG and free fatty acid levels by 32.05% and 23.67%, respectively. Further, RPs stimulated the increased expression levels of mRNAs (lecithin–cholesterol acyltransferase, adenosine triphosphate-binding cassette transporter A1, scavenger receptor class B type 1, and liver X receptor α) and hepatic lipolytic enzymes involved in HDL metabolism. These results suggest that RPs can help in preventing the occurrence of atherosclerosis. Further, a study involving 18 male adults has shown a significant increase in HDL-C levels (*p* = 0.047) and a decrease in serum uric acid (*p* = 0.030) levels during the rice endosperm protein (REP) intake period [132].

The digestibility of RPs is dependent upon the extraction method, which is lower with α-amylase (RP-E) compared to alkali (RP-A) [151]. The digestibility of RPs is crucial for the modulation of cholesterol absorption. RP-E-fed 7-week-old male Wistar rats exhibited lower levels of TC, TG, and fat deposits (both perirenal and epididymal) in comparison to casein and RP-A, both in plasma and liver. Further, RP-E significantly reduced the hepatic free and esterified cholesterol levels by 43.6% and 56.4%, respectively. The results suggest that RPs with lower digestibility levels inhibit cholesterol absorption (hypocholesteromic action).

### 6.3. Anti-Cancer Activity

Rice prolamin, a grain protein component, possesses an anti-leukaemia immune response [152]. Human peripheral blood mononuclear cells (PBMCs) from the blood of healthy subjects were separated and incubated. The PBMC-conditioned media (PBMC-CM) were prepared by incubating the cells with or without various rice proteins (albumin, globulin, glutelin, and prolamin). The PBMC-CM prepared from prolamin treatment exhibited a significant increase in the production of tumor necrosis factor-α (TNF-α). Additionally, prolamin-prepared PBMC-CM effectively inhibited the growth of U937 leukaemia cells and promoted the monocyte differentiation of U937 cells. Interestingly, antibody neutralization showed that the anti-leukaemia immune response caused by rice extract, endosperm extract, and prolamin was partially blocked indicating that prolamin is responsible for the anti-leukaemia immune effect.

Rice bran-derived pentapeptide (Glu-Gln-Arg-Pro-Arg; EQRPR) has shown anti-proliferative activity against human breast cancer cells, MCF-1, and MDA-MB-231 [153,154]. The pentapeptide (1000 μg/mL)-treated breast cancer cells showed a significant decrease in the survival rates compared to the control. The inhibitory effect of the pentapeptide was relatively more pronounced on MCF-7 cells (90.9%) compared to MDA-MB-231 cells (87.0%) at 72 h and 96 h, respectively [154]. Pentapeptide treatment of MCF-7 and MDA-MB-231 cells resulted in distinct changes in the morphology of these cells including cell floating and shrinkage, nucleic blebbing, and the presence of granular apoptotic bodies. These morphological changes observed after 72 h are classical features of apoptotic cells. Further, DNA fragmentation occurred in both breast cancer cell lines, a documented feature of cell death caused by apoptosis. Interestingly, the pentapeptide treatment (1000 μg/mL) of the breast cancer cells significantly increased the Caspase-3/7 activity levels. The impact was more noticeable at 96 h after treatment in MDA-MB-231 cells compared to MCF-7 cells. Caspase cascade is usually a sign of a cell undergoing apoptosis. The breast cancer cells when incubated with pentapeptide (1000 μg/mL) exhibited a significant down regulation of ‘Cyclooxygenase-2 [COX-2]’ (target for breast cancer treatment) in MCF-7 cells at 96 h. However, pentapetide treatment (1000 μg/mL) substantially increased the tumor protein (p53, a tumor suppressor) levels in both the breast cancer cells at 72 h and 96 h.

Glycoprotein extracted from rice bran (GRB) flour showed significant antitumor activity preventing the tumor metastasis in colon 26-M3.1 cells [155]. The protein and carbohydrate content of the glycoprotein was 55.8% and 5.1%, respectively [156]. The intravenous administration of colon 26-M3.1 cells with GRB (5 mg/kg) inhibited the lung tumor metastasis by 77.0% compared to the untreated mice. GRB-treated mice exhibited higher toxicity levels to YAC-1 cells compared to untreated mice indicating that the tumor inhibitory effect of GRB is through NK cell activation; NK cells suppress tumor growth and metastasis [157]. Further, the levels of granzyme B were significantly elevated (1.7-fold higher) in NK cells isolated from GRB-treated mice than those from normal mice. Activated macrophages release different cytokines that stimulate strong antitumor and antimetastatic responses [155]. Peritoneal macrophages produced different cytokines like TNF-α, IL-6, and IL-12, which play a role in the activation of immune system and antitumor activities.

Wattayagorn et al. [158] have demonstrated the effect of hydrolyzed rice bran protein extract (HRBE) on inducing apoptosis, senescence, and arrest of G1/S cell cycle in human colon cancer cell lines. The MTT assay showed that HRBE has greater cytotoxicity effects on metastatic cancer cell lines (SW-620, IC_50_ = 5468 μL/mL) compared to non-metastatic cancer cell lines (HT-29, IC_50_ = 6045 μL/mL) and fibroblast normal cells (PCS-201-010, IC_50_ = 6745 μL/mL) after 72 h. The results indicate that HRBE had a lower effect on normal cells compared to the cancer cell lines. Further, HRBE had a significantly higher senescence inductive effect on HT-29 cells (86% at 5 mg/mL) compared to SW-620 cells (32% at 5 mg/mL). HRBE had also an apoptotic inductive effect on the colon cancer cells (SW-620), and the morphology of the SW-620 cells changed with HRBE treatment. After 72 h of treatment with HRBE, cytoplasmic shrinkage, membrane blebbing, and chromatin condensation occurred, characteristic apoptotic-related features. The cellular apoptotic after 72 hrs of HRBE treatment at 10 mg/mL was 76% in the SW-620 cells. Interestingly, the HRBE treatment of HT-29 cells at 5 mg/mL and 10 mg/mL significantly decreased the percentage number of cells in S and G2/M cell phases while it increased in the G0/G1 cell phase. Further, it was shown that bioactive peptides (>50 kDa) exhibited higher growth inhibitory activity on SW-620 compared to other fractions, i.e., higher anti-cancer activity. The peptides (>50 kDa) might inhibit the colon cancer cell lines through the apoptosis-induced mechanism.

Inflammation is an important inducer of tumor progression, and it may cause different cancers [159]. Inflammation can also cause cancer cell proliferation, angiogenesis, and cell mobility, thus reducing inflammation that helps in treating cancer. In a study, the rice bran protein hydrolysates (RBP) alleviated the elevated levels of blood glucose, lipid, and insulin and restored insulin in high carbohydrate–high fat (HCHF) diet-fed rats. Further, RBP reduced the pro-inflammatory cytokine gene expression in rats fed on HCHF. RBP significantly reduced the expression levels of all the pro-inflammatory genes, viz., Interleukin-6 (*IL-6*), Tumor Necrosis Factor-*α* (*TNF-α*), Monocyte chemoattractant protein-1 (*MCP-1*), and Nitric Oxide Synthase (*NOS-2*). In addition, administering RBP to the rats resulted in the increase in the levels of interleukin-10 (*IL-10*), an anti-inflammatory gene [160].

## 7. Applications of Rice Grain Protein

In recent years, the food industry has been interested in rice protein as a sustainable, affordable, and high-quality source. It exhibits hypoallergenic, anti-inflammatory, antioxidative, and anti-hypertensive and hypoglycemic properties enabling it to replace soy, casein, and fenugreek [52,161]. It is the first solid food given/fed to infants because of its hypoallergenic property [22]. Rice protein-based formula is replacing cow’s milk and soy’s milk for children [162]. Rice grain, especially at a younger age, has a high protein content and digestibility, making it a major reason to replace other cereal and legume proteins [37]. Apart from children’s food, rice protein is a natural substitute for preparing gluten-free bakery products and beverages [161]. Additionally, manufacturers use rice protein concentrates as value-added ingredients in the production of edible films [163], meat extenders [164], and protein supplements for athletes [162]. Therefore, rice, in both its grain form and hydrolyzed protein concentrates, is establishing its position in the food industry.

## 8. Conclusions

Globally, the local and societal preferences decide the type and form of rice consumed. Despite wide variation in the consumption pattern of rice, it is an integral part of the human diet. Millions of people across Asia and Africa suffer from nutrient deficiencies (protein also) and NCDs. Interestingly, rice production and consumption is highest in these two continents. Therefore, nutrient-enriched rice varieties with high yielding ability will at least partially aid in addressing the issues of nutrient deficiency and NCDs. The recent COVID-19 pandemic created heightened awareness in people to adopt a healthy lifestyle including the consumption of nutrient-rich foods. International organizations like WHO and UNICEF work tirelessly for the improvement of dietary nutrition especially in vulnerable populations across the globe. Globally, rice could play a very important role in meeting the twin demands of food and nutritional security. Protein-rich rice can to a certain extent help in preventing life-threatening conditions like cardiovascular diseases (CVDs), cancer, and diabetes.

## Figures and Tables

**Table 1:** Global production of major cereals (million tonnes).

Region	Rice *	Wheat	Maize	Millet	Sorghum
Africa	42.76	26.34	95.00	13.13	25.95
Asia	716.77	352.34	403.73	17.31	7.67
Europe	3.33	269.26	119.03	0.66	1.03
Central America	1.37	3.47	31.75	-	5.05
North America	9.90	81.26	404.77	0.44	8.07
South America	24.19	24.68	186.27	0.01	7.18
Oceania	0.50	41.59	0.57	0.04	2.33

Source: [14] https://www.fao.org/faostat/en/#data/FBS (accessed on 9 January 2025; 2023 data); * Rice, paddy; -, data unavailable.

**Table 2:** Global per capita (kg/capita/yr) availability of major cereal food crops.

Region	Rice	Wheat	Maize	Millet	Sorghum
Africa	37.07	45.67	40.56	6.92	14.37
Asia	117.12	68.67	7.76	2.64	2.36
Europe	8.95	113.28	5.96	0.20	-
Central America	16.92	37.98	106.50	-	0.59
North America	12.20	90.95	12.01	-	0.23
South America	37.98	62.59	29.56	-	-
Oceania	28.24	74.70	3.07	-	0.51

Source: [14] https://www.fao.org/faostat/en/#data/FBS (accessed on 9 January 2025; 2022 data); -, data unavailable.

**Table 3:** Global protein supply (g/capita/day) from different sources.

Region	Plant	Meat	Eggs	Dairy	Fish and Seafood
Africa	50.61	7.83	0.70	3.53	2.57
Asia	58.83	15.30	3.65	7.15	6.78
Europe	44.22	32.54	4.25	23.02	6.10
Central America	42.89	29.65	5.57	10.25	3.51
North America	40.18	49.28	4.96	22.42	4.77
South America	38.71	36.21	3.74	11.64	2.69
Oceania	38.58	39.29	1.81	12.46	5.62

Source: [14] https://www.fao.org/faostat/en/#data/FBS (accessed on 9 January 2025; 2022 data).

**Table 4:** Global protein supply (g/capita/day) from different crops and their products.

Region	Rice	Wheat	Maize	Millet	Sorghum
Africa	4.81	10.86	7.69	1.51	3.34
Asia	15.42	18.66	1.16	0.62	0.54
Europe	1.04	25.14	0.94	0.04	-
Central America	2.14	7.89	18.51	-	0.15
North America	1.69	18.62	1.57	-	0.06
South America	5.00	13.42	4.99	-	-
Oceania	3.33	15.48	0.48	-	0.11

Source: [14] https://www.fao.org/faostat/en/#data/FBS (accessed on 9 January 2025; 2022 data); -, data unavailable.

**Table 5:** Amino acid composition in different types of rice.

Essential Amino Acid	Milled Rice [24]	Milled Rice [69]	Milled Rice [22]	Milled Rice [70]	Milled Rice [52]	Brown Rice [24]	Brown Rice Isolate [71]	Scented Rice (Milled) [72]
Histidine	2.3–2.7	2.4	2.5	1.18–2.53	1.19–3.49	2.4–2.6	1.6–1.8	2.2
Isoleucine	3.7–4.8	3.8	3.8	3.75–8.51	2.69–5.18	3.6–4.6	3.2–3.4	3.0
Leucine	8.4–8.6	8.2	8.2	1.68–4.19	5.30–9.51	8.3–8.9	6.2–6.4	7.5
Lysine	3.4–4.2	3.7	3.3	1.28–3.49	2.2–6.24	3.9–4.3	2.1–2.4	4.2
Cysteine	1.8–2.6	1.6	3.9	-	0.13–3.42	2.2–2.4	1.6–1.8	-
Methionine	2.3–3.0	2.1		0.16–2.25	0.65–3.49	2.3–2.5	2.2–2.8	2.7
Phenylalanine	5.3–5.5	4.8	10.1	2.31–5.71	3.5–6.30	5.0–5.3	4.1–4.4	5.2
Tyrosine	4.4–5.5	2.5		0.81–3.21	1.33–6.0	3.8–4.6	4.3–6.2	3.1
Threonine	3.7–3.9	3.4	3.5	1.98–4.92	2.09–5.06	3.9–4.0	2.8–3.0	4.0
Tryptophan	1.3–1.8	1.3	0.8	-	-	1.3–1.5	1.1–1.2	-
Valine	4.9–6.8	5.8	5.1	2.53–6.02	3.78–6.80	5.0–6.6	4.3–4.6	4.8

Note: values in (g/100 g); (-) data not available; numbers in [] indicate the references.

## Supplementary Materials

The following supporting information [32,70,102,103,104,105,106,107,108,109,110,111,113,114,115,116,117,118,119,121,126,127,128,165,166,167,168,169,170,171,172,173,174,175,176,177] can be downloaded at https://www.mdpi.com/article/10.3390/ijms26073163/s1.
