# Supplementary material for: From Gene to Plate: Molecular Insights into and Health Implications of Rice (Oryza sativa L.) Grain Protein
Source: Int J Mol Sci. 2025 Mar 29;26(7):3163. doi: 10.3390/ijms26073163 (PMC11989779; doi:10.3390/ijms26073163)
Supplement: Supplementary file 1 [file ijms-26-03163-s001.zip › ijms-3497818-supplementary.pdf]

Supplementary Table S1. QTLs identified for grain protein content, protein fractions, amino acids and protein index in rice.

S.No	Pedigree / Population type	QTL/Gene/Chromosome	Chromosome Interval / Marker	Morphology	Reference
			Protein content		
1	Aromatic germplasm (113)	QTL.pro.1	RM5	The QTL had a LOD 2.02 and explained 7.89% of variance.	[32]
2	Milyang 23 / Tong 88-7 (RILs)	qPC1.1, qPC1.2, qPC7.1	4632918-4708475: (qPC1.1)<br>39162572-39234399: (qPC1.2)<br>5627191-5803738: (qPC7.1)	➢ The three QTLs co-located with QTLs for Ser (*qAAC1.3*), His (*qAAC1.6*) and Thr (*qAAC7.2*), respectively.<br>*qPC1.2* explained 18.1% of the phenotypic variance, the largest additive effect.	[102]
3	Cheongcheong / Nagdong - (DHs)	qPC7	RM8261	➢ The *qPC7* was identified at the same flanking marker (RM8261) as for amylose content (*qAC7*).<br>➢ *qPC7* had 14% and 3.97 variance percentage and LOD value respectively.	[104]
4	Asominori/IR64 (CSSL)	qPC-8a, qPC-3, qPC-6, qPC-1a, qPC-1b, qPC-2, qPC-3, qPC-8b, qPC-11	G1149: (qPC-8a)<br>C563: (qPC-3)<br>C688: (qPC-6)<br>R1982: (qPC-1a)<br>XNpb113: (qPC-1b)<br>XNpb67: (qPC-2)<br>C1677: (qPC-3)<br>XNpb41: (qPC-8b)<br>C1350: (qPC-11)	➢ *qPC-8* was located repeatedly near the G1149 marker on chromosome 8 in six environments, with PVE of 28.9%.<br>➢ Near the G1149 marker on chromosome 8, one cluster harboring nine QTLs (*qPGWC-8*, *qACE-8*, *qAC-8*, *qPC-8a*, *qBDV-8a*, *qSBV-8b*, *qLT-8a*, *qTD-8a*, and *qIVOE-8a*) was found to control the nine rice quality traits.<br>➢ These nine QTLs were detected in four to eight environments, with mean PVEs of 13.7% to 36.0 indicating higher repeatability and a larger contribution rate to phenotypic variation.	[105]
5	Xieqingzao B / Milyang 46 (RILs)	qPC-3, qPC-4, qPC-5, qPC-6, qPC-10	RM251–RM282: (qPC-3)<br>RG214–RG620: (qPC-4)<br>RG470–RZ70: (qPC-5)<br>RM190–RZ516: (qPC-6)<br>RM184–RM3229B: (qPC-10)	➢ *qPC-6* was a major QTL located near the *Wx* marker RM190 on chromosome 6, explaining 19.3% of the phenotypic variance.<br>➢ The other four QTLs explained 3.9–10.5% of the phenotypic variance and had additive effects of 0.213– 0.343%.<br>➢ All the QTLs had the enhancing alleles from MY46.	[106]
6	Caiapo / IRGC 103544 (DHs)	Pro-1, Pro-2, Pro-6, Pro-11	RM226-RM297: (Pro-1)<br>RM6-RM112: (Pro-2)<br>RM190-RM253: (Pro-6)<br>RM209-RM229: (Pro-11)	➢ *Pro6* mapped to C962–Wx interval for protein content on chromosome 6 near the waxy locus.	[107]

7	Gui 630 / 02428 (DHs)	qRPC-1, qRPC-4, qRPC-5, qRPC-6, qRPC-7	RG811-BP127: (qRPC-1) C22-RG449d: (qRPC-4) RG435-RG172a: (qRPC-5) RG171-RG119a: (qRPC-6) ZG34B-G20: (qRPC-7)	➢ The five QTLs collectively explained 74% of the phenotypic variation. ➢ *qRPC-5* had the largest effect accounting for 35% of the phenotypic variation. ➢ *qRPC-7* explained 23% of the phenotypic variance.	[108]
8	Zhenshan97 / Minghui63 (RILs)	QTL on Chromosome 6 and 7	C952-Wx (on Chromosome 6) R1245-RM234 (on Chromosome 7)	➢ One QTL mapped in the interval of C952-Wx on chromosome 6, with PVE% of 13.0% and LOD of 6.8. ➢ The two QTLs explained 17.7% of the phenotypic variance with LOD=9.2.	[109]
9	9311/Nipponbare (CSSL)	qGPC2-1, qGPC2-2, qGPC2-3, qGPC2-4, qGPC3-1, qGPC3-2, qGPC4, qGPC5, qGPC6-1, qGPC6-2, qGPC7-1, qGPC7-2, qGPC8, qGPC9-1, qGPC9-2, qGPC10-1, qGPC10-2, qGPC10-3, qGPC11, qGPC12-1, qGPC12-2, qGPC12-3	RM5390-(qGPC2-1); RM3762-(qGPC2-2); RM191-(qGPC2-3); RM1342-(qGPC2-4); RM411-(qGPC3-1); RM3564-(qGPC3-2); RM6748; RM348-(qGPC4); RM289-(qGPC5); RM587-(qGPC6-1); RM3498-RM3628-(qGPC6-2); RM542-(qGPC7-1); RM1135-(qGPC7-2); RM339-(qGPC8); RM219-(qGPC9-1); RM566-(qGPC9-2); 10–1.63-(qGPC10-1); 10–9.12-(qGPC10-2); RM8201-(qGPC10-3); RM1812-(qGPC11); RM1261-(qGPC12-1); RM277-(qGPC12-2); RM1277-(qGPC12-3)	➢ A total of 22 QTLs across all the 12 chromosomes except chromosome 1 was detected. ➢ qGPC2-1, qGPC2-4, qGPC3-2, qGPC4, qGPC5, qGPC6-1, qGPC6-2, qGPC7-1, qGPC7-2, qGPC9-1, qGPC9-2, qGPC10-2 and qGPC11 were detected in both years of the study. ➢ qGPC6-1 was mapped to a QTL cluster where the Waxy gene is located.	[110]
10	YK17 x Hangzhou (RILs)	qPC1, qPC5, qPC11, qPC6, qPC7	RM8236–RM5536: (qPC1) RM17852–RM165: (qPC5) RM26998–RM27181: (qPC11) RM7158–RM3414: (qPC6) RM3859–RM11: (qPC7)	➢ The QTL *qPC6* detected repeatedly on the marker position near the Wx locus located on chromosome 6. ➢ *qPC1*, a major QTL for PC on chromosome 1 was detected under both growing seasons.	[111]
11	Xieqingzao B / Milyang 46 (RILs)	qPC-3, qPC-4, qPC-5, qPC-6, qPC-10	RM251–RM282: (qPC-3) RG214–RG620: (qPC-4) RG470–RZ70: (qPC-5) RM190–RZ516: (qPC-6) RM184–RM3229B: (qPC-10)	➢ *qPC-6* was a major QTL located near the *Wx* marker RM190 on chromosome 6, explaining 19.3% of the phenotypic variance. ➢ The other four QTLs explained 3.9–10.5% of the phenotypic variance and had additive effects of 0.213– 0.343%. ➢ All the QTLs had the enhancing alleles from MY46.	[106]
12	F_6 RILs (Samba Mahsuri x IR36ae)	*qGI2.1/qAC2.1* *qPC2.1*	The interval for *qPC2.1* is 10.13 – 10.33 Mb. The SNPs for *qPC2.1* are snp_02_10141997,	➢ The QTL *qGI2.1/qAC2.1* influences low GI and high Amylose ➢ The QTL *qPC2.1* influences high protein content. This region	[114]

			snp_02_10142042, snp_02_10142047, snp_02_10142051	contained candidate gene *LOC_Os02g17620* (isochorismatase) ➢	
13	Zhenshan 97B / Delong 208 (RILs)	qPr1, qPr2, qPr4,qPr7, qPr8, qPr9	RM493–RM562 : (qPr1) RM154–RM233A : (qPr2) RM349–MRG113:(qPr4) RM445–RM418 : (qPr7) RM149–RM433 : (qPr8) RM460–RM257 : (qPr9)	➢ Two QTLs (*qPr1* and *qPr7)* were detected in two different years. ➢ The allele of *qPr1* from Zhenshan 97B decreased the protein content whereas *qPr7* increased in milled rice. ➢ The PVE% varied from 5.1 to 25.9.	[116]
14	Koshihikari / Kasalath (BILs)	qPC-2, qPC-3, qPC-7, qPC-10	E61928S-C132: (qPC-2) R250-C746: (qPC-3) C847-C596: (qPC-7) C16-C809: (qPC-10)	➢ QTL *qPC-2* was consistently detected in two environments. ➢ QTL *qPC-7* had the highest LOD score and the larger effect was only significant in E3, accounting for PVE% of 12.11.	[118]
15	Zhonghua 11	*OsAAT1*, *OsAAT2*, *OsAAT3*, *EcAAT*	--	➢ The total leaf AAT activities in plants over-expressing *OsAAT1*, *OsAAT2*, and *EcAAT* were 26.6, 23.6, and 19.6 A/ min/ mg FW, which were signifcantly higher than that in the wild-type control (17.7 A min^{-1} mg^{-1} FW). ➢ Similarly transgenic plants of *OsAAT1*, *OsAAT2*, and *EcAAT* had 22.2, 21.1, and 11.1%, respectively higher protein contents than wild-type plants.	[119]
16	Hwayoung/Dongjin (Transgenics/Mutants)	*OsAsn1* (Chr 3)	The locus number for the *OsASN1* is Os03g0291500	➢ Grain protein content was enhanced in *OsAsn1* over expressing (OX) plants to 117% (OX1) and 114% (OX2) compared with that in WT. ➢ *OsASN1* is required for grain yield and grain protein contents under both N-sufficient (conventional paddy fields) and N-limiting conditions.	[121]
17	Nipponbare (Mutant lines)	*OsLHT1* (Chr 8)	The locus number for the *OsLHT1* is Os08g03350.1	➢ *Oslht1* seeds accumulated higher total N and proteins, total and individual free amino acids by ~30-35%. Knockout of *OsLHT1* decreased N uptake efficiency and physiological utilization efficiency by about 55% and 72%, respectively	[126]
18	Zhenshan 97 / Nanyangzhan (RILs)	qPC1/ *OsAAP6*	RM472-RM104	➢ Fine mapping of qPC1 locus revealed that it corresponds to *OsAAP6* (amino acid permease), highly expressed in seeds and belonging to *OsAAT* family in rice.	[127]

				➢ Higher expression of *OsAAP6* leads to higher GPC. *OsAAP6* greatly enhances root absorption of certain amino acids and also effects the distribution ➢ of various amino acids.	
19	ARC10075 / Naveen (BC_3F_4)	qGPC1.1, qSGPC1.1,qSGPC2.1, qSGPC7.1,qSGPC11.1	CSCWR_Os01g02590_61041 (qGPC1.1 and qSGPC1.1) CSCWR_Os02g10740_65058 (qSGPC2.1) SCR100_Os07g37440_17971 (qSGPC7.1) SCR200_Os11g07480_87716 (qSGPC11.1)	➢ Three QTLs, *qGPC1.1* (grain protein), *qSGPC2.1, qSGPC7.1* (both for single grain protein content) were stable over the environments with PVE of 13%, 14% and 7.8% respectively. ➢ Inside *qSGPC1.1* region, one gene (Os01g0111900) was found which encoded glutelin family protein. ➢ Similarly, *qSGPC7.1* was detected near to a cluster of genes encoding three peptidase proteins.	[128]
20	Two BC_3F_2 populations (Kongyu 131/TKM9 : P-I Kongyu 131/Bg 94-1: P-II)	Population I : *qGpc1.1, Gpc6.1, qGpc7.1, qGpc10.1* Population II : *qGpc3.1, qGpc4.1, qGpc7.2, qGpc10.2*	RM292-T1-3 (*qGpc1.1*) T6-7-T6-4 (*qGpc6.1*) T7-1-T7-3 (*qGpc7.1*) RM239-D10-7F (*qGpc10.1*) RM523-RM7 (*qGpc3.1*) RM551-4–5.8 (*qGpc4.1*) D7-2-RM418 (*qGpc7.2*) 10–2.5–10-12.0 (*qGpc10.2*)	➢ A total of 40 QTLs for five traits (Globulins, Prolamins, Albumins, Glutelins and GPC were detected ➢ Eight (8) QTLs were exclusively for GPC which covered the chromosomes 1, 3, 4, 6, 7, and 10 ➢ These detected QTLs were co-localized with previously reported QTLs for protein content ➢ The variance explained (R^2) ranged between 4.1 – 23-63 % ➢	[165]
21	F_2-NIL populations - Zhenshen 97/Delong 208 Zhenshen 97/Nanyangzhan	*qPC2.1, qPC7.1, qPC7.2* (ZS97B/NYZ) *qPC1.1* (ZS97B/DL208)	Chr.2 - RM555-RM492 (*qPC2.1*) Chr. 7 - RM125-RM214 (*qPC7.1*) Chr. 7 - RM1186-RM5499 (*qPC7.2*) Chr. 1 - RM493-RM562 (*qPC1.1*)	➢ The QTLs were validated and were co-localized with SNPs detected in GWAS study ➢ *qPC2.1* co-localized with sf0206873340, *qPC7.1* with sf0706126055, *qPC7.2* with sf0709202668 and *qPC1.1* with sf0113186602 ➢ The variance explained by these QTLs ranged between 9.82 to 43.4 %	[166]
22	Zhenshen 97/Nanyangzhan	*OsNAC74*	Chromosome 1	➢ OsAAP6 affects the grain protein content. The variation sites in OsAAP6 were located at -7 to -12 bp upstream ➢ *OsNAC74* acts as transcription factor to OsAAP6 regulating its expression, there by improving the grain protein content ➢ *OsNAC74* activity was validated by generating *Osnac74*	[167]

				mutants in which the downregulation of OsAAP6 was observed with decreased protein content but the grain yield was not affected	
23	A set of 274 diverse germplasm lines	qPC8.2<br>qPC1.2,<br>*qProt1*<br>qPC6.2	RM556 (qPC8.2; Chr. 8 : 22.3 Mb), RM220 (qPC1.2; Chr. 1 : 240 cM), RM5638 (*qProt1*; Chr. 1 : 20.9 Mb) and,<br>RM253 (qPC6.2; Chr. 6 : 5.4 Mb)	➢ A total of 4 QTLs were identified across chromosome 1, 6 and 8.<br>➢ Among them *qProt1* and qPC6.2 were detected in different studies. Hence, they are said to be validated and can be used in breeding program	[168]
24	Huanghuazhan / Jizi1560 (RILs)	qGPC1-1	JD1006–JD1075	➢ Genotyping-by-re-sequencing identified 14 QTLs in 2016 and 2017.<br>➢ Seven of the fourteen QTLs were stable and repeatedly identified across two years.<br>➢ *qGPC1*, *qGPC3-1* and *qGPC5* were commonly identified using both high- and low-density genetic maps.<br>➢ *qGPC1* identified in this study contributed 11.78% to 13.33% of phenotypic variations.	[169]
25	Landraces, cultivars and protein biofortified genotypes	qPC3, qPC3.1, qPC5.1, qPC6.1, qPC8, qPC9.1, qPC12.1	RM-7, RM6712 and *OsNAC*: (qPC3 and qPC3.1)<br>RM6209: (qPC5.1)<br>RM204: (qPC6.1)<br>RM407: (qPC8)<br>RM34: (qPC9.1)<br>RM260-RM309: (qPC12.1)	➢ Three novel QTLs *qPC3.1*, *qPC5.1* and *qPC9.1* were detected.<br>➢ Four reported QTLs *(qPC3, QPC8, qPC6.1 and qPC12.1)* were validated for use in breeding programs.<br>➢ Three QTLs *(qPC6, qPC6.1 and qPC6.2*) may be same QTL controlling PC in rice.<br>➢ The strongly associated markers with grain PC, namely *qPC3, qPC3.1, qPC5.1, qPC6.1, qPC8, qPC9.1 and qPC12.1,* will be useful for their pyramiding for developing protein rich high yielding rice.	[170]
26	Yukihikari / Joiku462 (RILs)	qPC1, qPC2, qPC3, qPC6.1, qPC6.2, qPC8, qPC12.1, qPC12.2	YJInDel-34–YJInDel-536_2: (qPC1)<br>YJInDel-61–YJInDel-67: (qPC2)<br>YJInDel-128–YJInDel-130: (qPC3)<br>YJInDel-197–YJInDel-206: (qPC6.1)<br>YJInDel-207–YJInDel-208: (qPC 6.2; 2014P)<br>YJInDel-208–YJInDel-218: (qPC 6.2; 2015P)<br>YJInDel-306–YJInDel-320: (qPC 8; 2014P)	➢ *qPC7* was detected under three environments with an average PVE% of ~10.5 and *qPC6.2*in two with an average PVE% of ~23.4<br>➢ *qPC1*, *qPC2* and*qPC6.1* are associated with the secondary effects of large biomasses (sinks and/or sources)<br>➢ *qPC3* is associated with the secondary effects of temperature during the filling period.	[171]

			YJInDel-321–YJInDel-324: (qPC 8; 2014S)<br>YJInDel-324–YJInDel-340: (qPC 8; 2015P)<br>YJInDel-1529: (qPC 12.1)<br>YJInDel-510–YJInDel-515: (qPC 12.2)	➢ *qPC6.2*, *qPC8* and *qPC12.2* are associated with the combined secondary effects of large biomasses and temperature.<br>➢ *qPC12.1* for PC itself.	
27	Cheongcheong / Nagdong (DHs)	qPro-2	RM12532–RM555	➢ The QTL was detected in the marker interval of RM12532-RM555 on chromosome 2 and had LOD score of 4.88.	[172]
28	Cheongcheong / Nagdong (DHs)	qPro-8, qPro-9, qPro-10	RM506-RM1235: (qPro-8)<br>RM219-RM23914: (qPro-9)<br>RM24934-RM25128: (qPro-10)	➢ The LOD scores were 2.57, 2.66, and 6.13 respectively.	[173]
29	Asominori / IR24 (RILs)	qPC-1, qPC-3.1, qPC-3.2, qPC-3.3, qPC-4, qPC-6, qPC-7.1, qPC-7.2, qPC-8, qPC-9, qPC-10, qPC-12	R886-R1485: (qPC-1)<br>XNpb212-G1318: (qPC-3.1)<br>R758-XNpb15: (qPC-3.2)<br>C606-XNpb238: (qPC-3.3)<br>R1854-R2373: (qPC-4)<br>C1003-C688: (qPC-6)<br>XNpb338-C796: (qPC-7.1)<br>XNpb268-R411: (qPC-7.2)<br>C483-C259G: (qPC-8)<br>R265B-XNpb36: (qPC-9)<br>C16-C809: (qPC-10)<br>XNpb24-C562: (qPC-12)	➢ Ten unconditional QTLs significantly influencing PC were identified at four stages (7, 14, 21, 28 DAF).<br>➢ Three QTLs were detected at 28DAF: *qPC-1*, *qPC-3.1*, and *qPC-8*.<br>➢ *qPC-8* was also detected at 21 DAF.<br>➢ *qPC-6* was detected at 7 and 14 DAF.<br>➢ Totally six genomic regions with conditional QTLs significantly affected PC at different stages.<br>➢ *qPC-7.2* accounted for 23.7% of the total variation 28 DAF, having the larger effect.<br>➢ *qPC-9* was expressed at 7 and 14 DAF.	[174]
30	Samgang / Nagdong (DHs)	qPC1.1, qPC11.1, qPC11.2	1008-RM575: (qPC1.1)<br>RM3428-11025: (qPC11.1; 2005)<br>11027-RM287: (qPC11.1; 2004)<br>RM287-RM26755: (qPC11.2)	➢ The *qPC11.2* was repeatedly detected across two years.<br>➢ Seven pairs of epistatic loci were identified on eight chromosomes for protein content and collectively explained 39.15% of phenotype variation.	[175]
31	Chuan7 / Nanyangzhan (RILs)	qPC-6, qPC-7	RM588–RM540: (qPC-6)<br>RM5436–RM6776: (qPC-7)	➢ Two QTLs collectively explained 7.19% of the variance.	[176]
			Amino acids		
32	Dasanbyeo / TR22183 (RILs)	qAla3, qVal3, qLeu3, qIso3, qPhe3, qLys3	id3015453-id3016090 (qAla3, qVal3, qLeu3, qIso3, qPhe3)<br>id3001422-fd10 (qLys3)	➢ The phenotypic variation ranged from 10.2 to 12.8%.<br>➢ Since multiple QTLs displayed a similar limit of detection plot, it could be assumed that a common QTL exhibited a pleiotropic effect on five of these amino acids.	[70]
33	Milyang 23 / Tong 88-7 (RILs)	qAAC1.1, qAAC1.2, qAAC1.3,	2886294-3539052: (qAAC1.1)<br>4301442-4494576: (qAAC1.2)<br>4632918-4708475: (qAAC1.3)	➢ QTLs were identified for all amino acids except for proline in this study.	[102]

		qAAC1.4, qAAC1.5, qAAC1.6, qAAC2.1, qAAC2.2, qAAC3.1, qAAC3.2, qAAC6.1, qAAC6.2, qAAC7.1, qAAC7.2, qAAC7.3, qAAC8.1, qAAC8.2,	5154287-5197935: (qAAC1.4) 37112359-37711021: (qAAC1.5) 39162572-39234399: (qAAC1.6) 4065908-4220624: (qAAC2.1) 18092703-18131695: (qAAC2.2) 4542873-4614153: (qAAC3.1) 5140419-5225129: (qAAC3.2) 3515361-3658340: (qAAC6.1) 4192641-4230522: (qAAC6.2) 4856196-5206110: (qAAC7.1) 5627191-5803738: (qAAC7.2) 11635687-14689865: (qAAC7.3) 19156876-26250116: (qAAC8.1) 26398897-26510920: (qAAC8.2)	➢ Five QTLs (*qAAC6.1*, *qAAC6.2*, *qAAC7.1*, *qAAC7 .2*, and *qAAC8.2*) were linked with the essential amino acid content. ➢ *qAAC6.1* and *qAAC7* were the two major QTLs that affected several AACs simultaneously. ➢ The combination of *qAAC6.1*^{M23} / *qAAC7.1*^{T887} exhibited significantly higher contents of 11 AAs (yr, Leu, Thy, Phe, Ser, Val, Ala, Ile, Arg, Asx, and Glx) than the allelic combinations of both parents (*qAAC6.1*^{M23} / *qAAC7.1*^{M23} and *qAAC6.1*^{T887} / *qAAC7.1*^{T887}). ➢ Maximum number of QTLs was detected for serine, a total of six loci including *qAAC1.3*, *qAAC1.4*, *qAAC1. 5*, *qAAC2.1*, *qAAC2.2*, and *qAAC7.1*.	
34	Zhenshan 77 / Milyang 46 (RILs)	qLys1.1, qLys1.2, qLys6, qLys9, qLys12, qCys7, qCys9, qCys12, qMet6, qMet9, qMet11	RG532-RM151: (qLys1.1) RG381-RG236 : (qLys1.2) RM225-RM6917: (qLys6) RM242-RM108 : (qLys9) RM20-RG81 : (qLys12) RZ721-RZ395 : (qCys7) RM105-RM3700 : (qCys9) RG81-S13126 : (qCys12) RM190- RZ516 : (qMet6) RZ698-RM296 : (qMet9) RZ816-RM332 : (qMet11)	➢ A total of 73 QTLs were detected for 17 amino acids in unmilled rice ➢ For three years, the R^2 ranged between 6.6 – 10.8 % (Lys), 6 – 16.5 % (Cys) and 7.2 -17.2 % (Met) ➢ It is also observed that the largest QTL cluster (14 QTLs) was located in RM190 – RM6917 region of short arm of chr. 6. Interestingly this region covers two florigen genes *RFT1* and *Hd3a* and stated that *RFT1* has strong and stable influence on most of the AAC in unmilled rice	[103]
35	387 rice accessions (244 – *indica*, 143 – *japonica*)	*LOC_Os07g20544*	11,864,886 – 11,951,886 bp (chromosome 7)	➢ *LOC*_Os07g20544encodes Aspartokinase (AK) protein	[115]
36	Zhenshan 97B / Delong208 (RILs)	qAa1, qAa7, qAa9	RM493–RM562 : (qAa1) MRG186–MRG4499 : (qAa7) RM328–RM107 : (qAa9)	➢ A total of 48 and 64 QTLs were detected for amino acid content in 2004/2005, respectively. ➢ Most QTLs co-localized, forming 12 QTL clusters on the chromosomes, and three QTL clusters (*qAa1*, *qAa7*, *qAa9*) were detected in both years. ➢ The Zhenshan97B alleles of *qAa1* and *qAa9* decreased the amino acid content whereas the Zhenshan 97B allele of qAa7 increased in milled rice.	[116]
37	Zhenshan 97 / Nanyangzhan (RILs)	QTL in chromosome 1	RM472-RM104 - (Chr 1) RM125-RM542 - (Chr 7)	➢ Totally, 18 QTL clusters for 19 components of amino acid content	[117]

		QTL in chromosome 7		were identified, 10 in 2002 and 6 in 2004.<br>➢ Two major QTL clusters in RM472-RM104 and RM125-RM542 were detected in two years. The PVE% of these two QTL was large, with ~30% in 2002 and ~40% in 2004.<br>➢ Of all the 18 QTL (clusters), 12 correspond to the loci involved in amino acid metabolism pathways.	
38	Zhonghua 11	*OsAAT1*, *OsAAT2*, *OsAAT3*, *EcAAT*	--	➢ The total leaf AAT activities in plants over-expressing *OsAAT1*, *OsAAT2*, and *EcAAT* were 26.6, 23.6, and 19.6 A/ min/ mg FW, which were signifcantly higher than that in the wild-type control (17.7 A min^{-1} mg^{-1} FW).<br>➢ The amino acid content in seeds of transgenic plants over-expressing *OsAAT1*, *OsAAT2*, and *EcAAT* was 16.1, 12.0, and 5.4% higher, respectively, than that in the control plants.	[119]
			Protein Fractions		
39	YK17 x Hangzhou (RILs)	qGLU4, qGLU5, qGLU6, qGLU8, qGLU10	RM17303–RM17377 : qGLU4<br>RM1024–RM18053 : qGLU5<br>RM7158–RM3414 : qGLU6<br>RM3702–RM310 : qGLU8<br>RM228–RM590 : qGLU10	➢ qGLU6 was detected repeatedly along the same genomic region as qPC6 and high correlation was observed between PC and GLU.	[111]
		qGLO1, qGLO5, qGLO6, qGLO10 qGLO11 qGLO12	RM6738–RM3738 : qGLO1<br>RM18457–RM163 : qGLO5<br>RM190–RM276 : qGLO6<br>RM1375–RM228 : qGLO10<br>RM26652–RM27181 : qGLO11<br>RM1880–RM5927 : qGLO12	➢ qGLO1 was detected as a major gene for GLO under all three population growing seasons.	
		qALB3, qALB4, qALB6, qALB10	RM282–RM3646 : qALB3<br>RM3529–RM18053 : qALB5<br>RM7158–RM190 : qALB6<br>RM160–RM205 : qALB9<br>RM7492–RM311 : qALB10	➢ qALB6 was found as a major determinant for ALB under all three population growing seasons with same marker interval of qPC6 and qGLU6.<br>➢ They also found that 3.3 kb *Wx* pre-mRNA is positively correlated with ALB.	
		qPRO1, qPRO2, qPRO4, qPRO6, qPRO8, qPRO12,	RM600–RM3341 : qPRO1<br>RM5812–RM3763 : qPRO2<br>RM7585–RM6659 : qPRO4<br>RM7158–RM3414 : qPRO6<br>RM3702–RM310 : qPRO8<br>RM5927–RM5746 : qPRO12	➢ qPRO6 QTLs were detected for the PRO protein and it shares the same genomic region as qPC6, qGLU6 and qALB6, indicating the influence of *Wx* gene region to control PRO.	
40	Asominori / IR24 (RILs)	qALB-1, qALB-2	R3203eXNpb113 : (qALB-1)<br>XNpb349eV83B : (qALB-2)	➢ *qALB-1* had large effect explaining 19.4% of the phenotypic variance. The allele from Asominori	[113]

				increased albumin content by 4.4% at this locus.<br>➢ *qALB-2* explained 9.1% of the variance. The IR24 allele at this locus increased albumin content by 3.0%.	
		qGLB-1, qGLB-2.1, qGLB-2.2, qGLB-5	XNpb113eXNpb93 : (qGLB-1)<br>XNpb89-3eC1470 : (qGLB 2.1)<br>XNpb250eC560 : (qGLB 2.2)<br>XNpb81eG1103 : (qGLB-5)	➢ The QTL *qGLB-1* had PVE% of 18.1 and itwas flanked by the same marker, XNpb113 for *qALB-1*.<br>➢ *qGLB-2.1*, *qGLB-2.2* and *qGLB-5* explained 13.4%, 10.6% and 18.0% of the phenotypic variance respectively.	
		qPLA-1, qPLA-3, qPLA-10	R210eC1211 : (qPLA-1)<br>XNpb48eC393B : (qPLA-3)<br>C16eC797 : (qPLA-10)	➢ *qPLA-10*is a major QTL with the largest effect (23.3%) and was mapped in the interval C16-C797 on chromosome 10.<br>➢ *qPLA-1* and *qPLA-3* explained about 16.4% and 11.5% phenotypic variance respectively.	
		qGLT-2, qGLT-10, qGLT-11, qGLT-12	XNpb89-3eC1470 : (qGLT-2)<br>C16eC797 : (qGLT-10)<br>XNpb320eC496 : (qGLT-11)<br>XNpb193eC562B : (qGLT-12)	➢ *qGLT-10* (PVE% of 14.8) for was located in the same region as qPLA-10.<br>➢ Interestingly*qGLT-2* was mapped in the same interval as *qGLB2.1*.	
41	Two BC_3F_2 populations (Kongyu 131/TKM9 : P-I Kongyu 131/Bg 94-1: P-II)	**Population I:**<br>*qAlb1.1, qAlb2.1, Alb2.2, qAlb4.1, qAlb6.1, qAlb10.1, qAlb11.1, qGol2.1, Gol3.1, qGol6.1, qGol 11.1, qPro2.1, qPro6.1, qPro11.1, qGlu2.1, qGlu3.1, qGlu10.1, qGlu11.1*	RM414-C1.43.1 (*qAlb1.1*); RM279-RM71 (*qAlb2.1*); RM263-RM526 (*qAlb2.2*); RM470-RM567 (*qAlb4.1*); RM111-RM402 (*qAlb6.1*); RM474-C10.13.5 (*qAlb10.1*); C11.24.4-RM224 (*qAlb11.1*); RM279-RM71 (*qGol2.1*); RM520-RM448 (*Gol3.1*); RM111-RM402 (*qGol6.1*); C11.24.4-RM224 (*qGol 11.1*); RM262-RM526 (*qPro2.1*); RM111-RM402 (*qPro6.1*); C11.22.5-C11.24.4 (*qPro11.1*); RM341-RM327 (*qGlu2.1*); RM448-RM442 (*qGlu3.1*); RM474-C10.13.5 (*qGlu10.1*); C11.24.4-RM224 (*qGlu11.1*)	➢ A total of 40 QTLs for five traits (Globulins, Prolamines, Albulins, Glutelins and GPC were detected<br>➢ For all the 4 protein fractions, a number 18 of QTLs were detected in P-I and 14 QTLs were detected in P-II<br>➢ All the detected QTLs were co-localized with previously reported QTLs<br>➢ The explained variance (R^2) ranged between 7.23 – 51.49 %	[165]
		**Population II :**<br>*qAlb2.3, qAlb2.4, qAlb7.1, qAlb7.2, qAlb8.1, qGol2.2, qGol2.3, qGol10.1, qPro1.1, qPro2.2, qPro5.1, qPro8.1, qGlu1.1, qGlu7.1*	RM145-RM301 (*qAlb2.3*); RM112-A2.35.29 (*qAlb2.4*); RM481-H7.9.2 (*qAlb7.1*); RM436-RM427 (*qAlb7.2*); RM506-8–8.1 (*qAlb8.1*); RM450-RM112 (*qGol2.2*); RM112-A2.35.29 (*qGol2.3*); 10–14.0–10-17.5 (*qGol10.1*); 1–9.0-RM297 (*qPro1.1*); RM112-A2.35.29 (*qPro2.2*); D5-5-RM509 (*qPro5.1*);		

			RM506-8–8.1 (*qPro8.1*); RM595-RM414 (*qGlu1.1*); D7-2-RM418 (*qGlu7.1*)		
42	*O. sativa* landraces (527)	SNPs on chromosomes 5 and 7	sf0710433511(Alb) sf0514987630 (Pro), sf0519612378 (Pro), sf0705739605 (Pro), sf0705159012 (Pro), sf0706210463 (Pro), sf0705534834 (Pro), sf0706363663 (Pro)	➢ GWAS study resulted with 107 trait associations, among them SNPs detected in different populations were mentioned<br>➢ The explained variance PV (%) was >10% for each SNP	[177]
			Protein index		
43	Koshihikari / Kasalath (BILs)	qPI-3.1, qPI-3.2, qPI-6.1, qPI-6.2, qPI-6.3, qPI-7, qPI-9, qPI-10	S879-S10251 : (qPI-3.1) R250-C746 : (qPI-3.2) R1952-G200 : (qPI-6.1) R2171-S2539 : (qPI-6.2) C358-C556 : (qPI-6.3) C847-C596 : (qPI-7) R2638-C1263 : (qPI-9) C16-C809 : (qPI-10)	➢ QTLs qPI-3.1 and qPI-7 were identified under three different environments, QTL qPI-3.2 in two environments and others in only one.<br>➢ The PVE% varied from 6.07 to 25.73. The highest PVE% was by qPI-7 under environment 3 (E3).<br>➢ QTL qPI-6.1 was linked with *Wx* locus.	[118]
44	Asominori / IR24 (RILs)	qPI-1, qPI-2, qPI-3, qPI-4, qPI-6, qPI-7.1, qPI-7.2, qPI-8, qPI-9.1, qPI-9.2, qPI-10, qPI-11, qPI-12	R2159-XNpb368 : (qPI-1) XNpb45-G1314B : (qPI-2) C1677-XNpb51 : (qPI-3) R1854-R2373 : (qPI-4) C962-XNpb170 : (qPI-6) XNpb338-C796 : (qPI-7.1) XNpb268-R411 : (qPI-7.2) C483-C259G : (qPI-8) R265B-XNpb36 : (qPI-9.1) C609-XNpb108 : (qPI-9.2) R1877-R844B : (qPI-10) C718-R1466 : (qPI-11) XNpb148-XNpb258 : (qPI-12)	➢ 11 unconditional QTLs for PI were detected.<br>➢ Three QTLs were detected at 28 DAF: *qPI-2*, *qPI-9.2*, and *qPI-12*.<br>➢ QTL *qPI-9.2* was also detected at 7 and 21 DAF, indicating that the accumulated effects of this QTL were too small to be detected at 14 DAF.<br>➢ Nine conditional QTLs were mapped to eight chromosomes.<br>➢ *qPI-11* was detected at 7 and 14 DAF but, it manifested opposite effects between those times.<br>➢ Using a conditional method, we also detected unconditional QTL, *qPI-8* at two stages.	[174]

Note: DHs – double haploids; RILs – recombinant inbred lines; BILs – backcross introgression lines; CSSL – chromosome substitution segment lines.
